# Practical Epoxidation of Olefins Using Air and Ubiquitous Iron-Based Fluorous Salen Complex

**DOI:** 10.3390/molecules29050966

**Published:** 2024-02-22

**Authors:** Yamato Kato, Miho Kanoh, Hina Kobayashi, Takayuki Shioiri, Masato Matsugi

**Affiliations:** Faculty of Agriculture, Meijo University, 1-501 Shiogamaguchi, Tempaku-ku, Nagoya 468-8502, Japan; 233561503@ccmailg.meijo-u.ac.jp (Y.K.); shioiri@mb.ccnw.ne.jp (T.S.)

**Keywords:** air, iron, fluorous, salen complex, epoxidation, olefin

## Abstract

The epoxidation of olefins by substituting “air” for potentially harmful oxidants was achieved using an oxidation method that integrated a fluorous iron(III) salen catalyst derived from common metals and pivalaldehyde. Several aromatic disubstituted olefins were converted into their corresponding epoxides with high efficiency and quantitative yields. This reaction represents an environmentally friendly oxidation process that utilizes an abundant source of air and employs a readily available metal, iron, in the form of salen complexes, making it an environmentally conscious oxidation reaction.

## 1. Introduction

Epoxide compounds are widely used as intermediates in pharmaceutical synthesis and functional materials [1,2]. Oxidants are required to oxidize olefins to epoxides, and air is the safest and cheapest oxidant for this purpose. To date, oxidation reactions employing molecular oxygen as an oxidant for the epoxidation of olefins, in conjunction with aldehyde reagents, have been reported [3,4,5,6,7,8,9,10,11,12,13,14,15,16].

In 1992, Kaneda et al. successfully conducted the oxygen epoxidation of olefins under oxygen-bubbling conditions using pivalaldehyde as a co-oxidant [3]. This study is significant because of its adoption of an environmentally friendly oxidation method featuring reagents with low explosiveness and no generation of byproducts from the oxidants. However, the continuous bubbling of oxygen, which is the source of oxygen, is required, making the operation cumbersome. Moreover, the use of high-concentration oxygen poses a potential risk of dust explosions. Therefore, the practical application of this method to plant-scale operations in an oxygen atmosphere is challenging. To enhance its applicability to industrial processes as an oxidant, there is a preference for utilizing the more readily available and safer “air directly” in large-scale synthesis.

Furthermore, Mukaiyama et al. presented a method for the asymmetric epoxidation of olefins using oxygen (not air) as the oxidant, combining a manganese salen complex [17,18] with pivalaldehyde [12]. However, this oxygen oxidation reaction requires the use of the rare metal manganese. Although there have been a few reports on the epoxidation of olefins using “air” as an oxidant, an efficient epoxidation reaction without using rare metals has rarely been reported.

In our previous study, we developed an asymmetric oxidation catalyst by introducing a fluorous tag at the 3,3′-position of the salen ligand. Using this complex, we achieved the asymmetric epoxidation of olefins under hypervalent iodine conditions for the first time using “iron” [19]. However, in this method, the use of oxidants, such as iodosobenzene was essential. If a reaction system using air and a common metal–iron complex can be realized, it has the potential to be applied to industrial processes as a safe and environmentally friendly synthetic method. Fluorous iron salen complexes have been suggested to have a different steric environment than normal salen complexes [19]; therefore, different and unique reactivities are expected. In this paper, we report the air epoxidation of olefins using a novel fluorous iron(III) salen complex with isoaldehyde as a co-oxidant. The reaction system using air directly as the oxidant is fundamentally different from using oxygen molecules, as it eliminates the need for the pre-preparation of oxidants. This enables the construction of an environmentally friendly and energy-efficient reaction system.

## 2. Results and Discussions

**Scheme 1:**
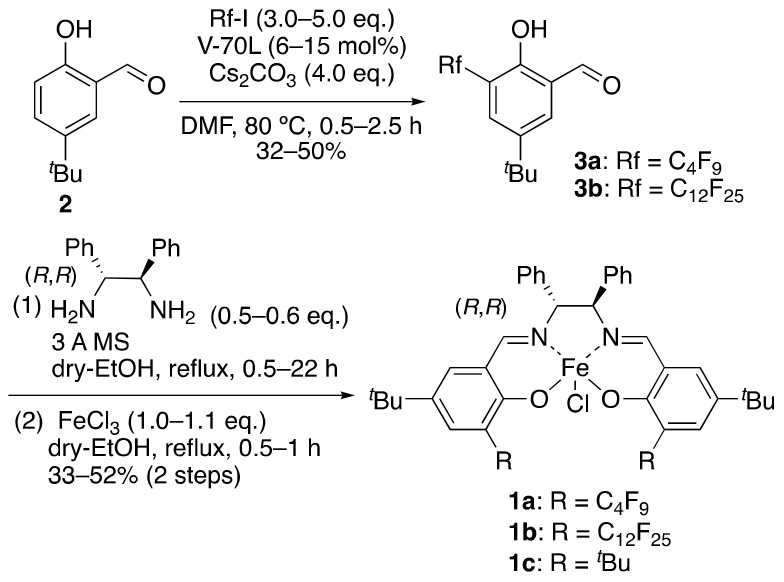
Synthesis of fluorous and non-fluorous iron(III) salen complexes **1a**, **1b**, and **1c**.

Two types of iron salen complexes with perfluoroalkyl groups of different lengths were synthesized based on the impact of variations in full-length fluorous-tagged iron salen complexes on catalytic activity. Fluorous iron(III) salen complex **1a** with a C_4_F_9_ tag at the 3,3′-position of the ligand was prepared using our previously reported procedure [20]. Complex **1b** with a C_12_F_25_ tag at the same position was also synthesized. In other words, perfluoroalkylation reactions were initiated from 5-*tert*-butylsalicylaldehyde **2** using V-70L [2,2′-azobis(2,4-dimethyl-4-methoxyvaleronitrile)] to obtain the perfluoroalkyl precursors **3a** and **3b**. Subsequently, each perfluoroalkyl precursor underwent condensation reactions with (1*R*,2*R*)-(+)-1,2-diphenylethylenediamine to form a salen ligand. The final step involved coordination exchange with iron(III) chloride to synthesize the desired fluorous iron(III) salen complexes, **1a** and **1b** (Scheme 1). Non-fluorous catalyst **1c** was synthesized using a known synthetic method in which the central metal of the Jacobsen-type Mn salen complex [17], with the R group being the *^t^*Bu group, was replaced with iron.

The epoxidation of olefins using air as the oxidant was investigated using the synthesized complexes **1a** and **1b**. The reaction was performed for 5 h at room temperature in acetonitrile using triphenylethylene **4a** as a substrate in the presence of an iron(III) salen complex and pivalaldehyde (Table 1) [12]. The reaction with complex **1a** yielded the desired epoxide **5a**; however, the conversion was only 73%. Notably, the reaction with complex **1b** yielded **5a** quantitatively with significantly better reactivity than that with **1a**. Even when the amount of **1b** was reduced from 1 to 0.5 mol%, no decrease in the reactivity was observed, and the target product was successfully obtained quantitatively. However, as in a previous report [19], chirality was not induced in this reaction, and the resulting product was racemic. Even when using non-fluorous catalyst **1c** under the same conditions, the reaction proceeded; however, its catalytic activity was low. These results showed that **1b** is the optimal catalyst for the air oxidation reaction among these catalysts.

Next, the aldehyde structure was added as a co-oxidant in the reaction, and complex **1b** was optimized (Table 2). Relatively good results were obtained when isovaleraldehyde was used; however, the yield was slightly lower than that when pivalaldehyde was used under the same conditions (Table 2, entries 1 vs. 2). When other aliphatic and alicyclic aldehydes were added, little progress was observed (Table 2, entries 3–5). Similarly, when aromatic aldehydes with nitro, methoxy, or methyl groups at the para-position, 2-naphthalaldehyde or heteroaromatic aldehydes, were used, the reaction did not proceed (Table 2, entries 6–12). These results showed that pivalaldehyde, which is used in combination with salen–manganese complexes, has been reported in the literature [12] and is the most suitable aldehyde as a co-oxidant in this reaction.

We then investigated the solvent effect on this reaction (Table 3). In addition to the successful progression observed in dry MeCN, the reaction proceeded smoothly when the protonic polar solvent, *^t^*BuOH, was used. However, at the 5-h mark, a thin-layer chromatography (TLC) test showed that the starting material had not disappeared; therefore, the starting material had to be fully consumed for 8 h (Table 3, entry 2). When dry MeOH was used, complex **1b** and the substrate did not dissolve in the solvent, leading to a significant reduction in the reaction yield (Table 3, entry 3). For non-protonic polar solvents, dry DMF and dry THF were considered, but neither showed significant progress in the reaction (Table 3, entries 4 and 5). When halogenated solvents, such as dry DCM and 2,3,4,5,6-pentafluorotoluene, were used, longer reaction times were required, but high conversion rates were achieved (Table 3, entries 6 and 7). These results showed that dry MeCN was the optimal solvent for this reaction.

Next, the substrate scope of this epoxidation reaction was investigated (Table 4). Quantitatively corresponding epoxides were obtained by extending the reaction time to 6 or 7 h using disubstituted internal olefins **4b** and **4c**, as well as terminal olefin **4d** as substrates (Table 4, entries 2–4). Interestingly, only an isomerized *trans*-type epoxide was produced in the reaction using *cis*-β-methylstyrene **4c** as the substrate, and no *cis*-type epoxide was observed. When substrates **4e**–**g** with electron-donating or fluorine substituents were used, the reactions proceeded smoothly and quantitatively to produce the corresponding epoxides (Table 4, entries 5–7). However, when (*E*)-2-styrylnaphthalene **4h** was used as the substrate, a decrease in reactivity was observed, and the conversion rate remained at 52% even when the reaction time was extended to 24 h (Table 4, entry 8). When substrate **4i** containing both trisubstituted and monosubstituted olefinic moieties was used, only the trisubstituted olefinic site underwent selective epoxidation with perfect regioselectivity, leading to the quantitative formation of monoxide **5i** (Table 4, entry 9).

**Scheme 2:**
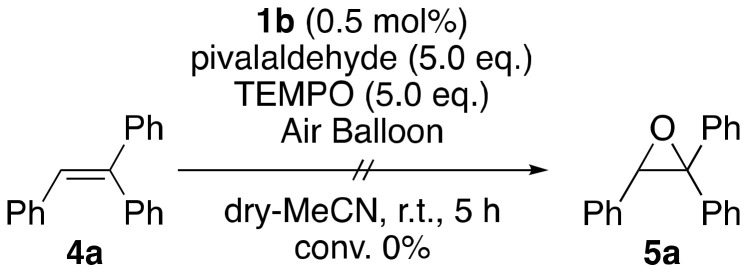
Control experiment using a radical scavenger.

In 2008, Köckritz et al. reported the epoxidation of olefins using aldehydes as co-oxidants and highlighted the involvement of radical intermediates in the reaction [5]. Notably, in the reaction using salen iron complex **1b**, isomerized trans epoxide was observed when *cis*-β-methylstyrene **4c** was used as the substrate (Table 4, entry 3). This observation indicated that the reaction also progressed through the radical species. Therefore, we conducted a control experiment by adding 2,2,6,6-tetramethyl-1-piperidinyloxy (TEMPO) as the radical scavenger (Scheme 2). Thus, the reaction did not proceed at all, suggesting that it also proceeds via radical species.

**Scheme 3:**
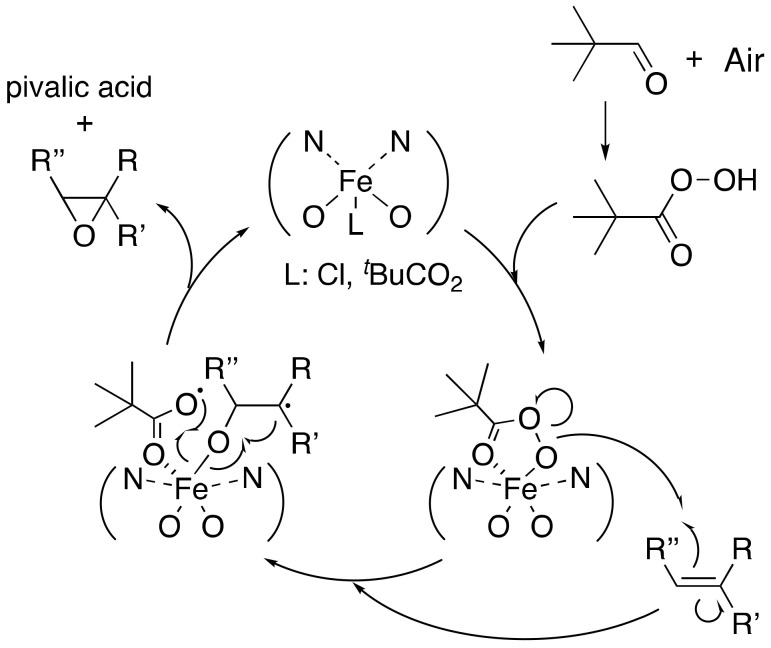
Plausible reaction mechanism.

It is known that pivalaldehyde forms a peroxyl radical species upon interaction with oxygen molecules [4,5,6]. The corresponding *trans* isomer was also generated in our reaction when *cis*-olefin was used as the substrate. These observations show that the peroxyl radical species interacts with iron, and the reaction may proceed through an iron(III) complex of radical species, as shown in Scheme 3.

## 3. Conclusions

Fluorous salen complex **1b**, which features iron as the central metal, was used as a catalyst to achieve the epoxidation reaction of olefins using “air” as the oxidizing agent. This reaction can be applied to various polysubstituted olefins to obtain the corresponding epoxides in high yields. This is an extremely safe reaction system that uses inexhaustible “air” as the oxidant. It is also an environmentally friendly oxidation reaction that uses iron, which is a common metal. In future studies, we aim to expand the catalytic reaction system to recycle and reuse fluorous salen complexes.

## 4. Experimental Section

### 4.1. Materials and Reagents

All the laboratory chemicals were purchased from Tokyo Chemical Industry Co., Ltd. (Tokyo, Japan), FUJIFILM Wako Pure Chemical Corporation (Richmond, VA, USA), Sigma-Aldrich Co., LLC (St. Louis, MO, USA), and Kanto Chemical Co., Inc. (Tokyo, Japan) and used without further purification unless otherwise stated. Solvents were removed using rotary evaporation under reduced pressure using a water bath at 50 °C. Nonvolatile compounds were dried in vacuo at 0.01 mbar. All reactions were stirred magnetically and monitored using thin-layer chromatography using silica gel plates. Purification by chromatography was performed on silica gel 60 N (spherical, neutral, 63–210 µm, Kanto Chemical Co., Inc.). Fluorous solid phase extraction (FSPE) was performed on FluoroFlash^®^ SILICA (40 µm, 60 A, Sigma-Aldrich, USA).

### 4.2. Analytical Instruments

Melting points were determined in ATM-01 of AS ONE Corporation and are uncorrected. Nuclear magnetic resonance (NMR) spectra were recorded using JNM-ECZ400S (^1^H: 400 MHz, ^13^C: 101 MHz, ^19^F: 376 MHz) spectrometers. ^1^H NMR spectral data are reported as follows: chemical shift in ppm referenced to TMS (δ 0.00 ppm), multiplicity (s = singlet, d = doublet, t = triplet, q = quartet, m = multiplet or unresolved, dd = double doublet, br s = broad signal), coupling constant (*J*: units in Hz), and integration. ^13^C NMR spectral data are reported as chemical shifts in ppm referenced to residual solvent (CDCl_3_ δ 77.16 ppm). ^19^F NMR spectral data are reported as follows: chemical shift (uncorrected), integral. High-performance liquid chromatography (HPLC) analyses were performed using DAICEL CHIRALCEL OD-3 column with UV Detector SPD-20A. High-resolution mass spectra were obtained using Exactive^TM^ Plus Orbitrap (Thermo Fisher Scientific, Waltham, MA, USA). The spectra were calibrated with Pierce^TM^ LTQ Velos ESI Positive Ion Calibration Solution before data acquisition.

### 4.3. General Procedure for the Epoxidation of Olefins Using Air

Under air atmosphere (vide Appendix A), pivalaldehyde (109 µL, 1.0 mmol, 5.0 eq.) was added to a suspension of olefin (0.2 mmol, 1.0 eq.) and Fe(III) salen complex (1.0 μmol, 0.5 mol%) in dry-MeCN (2 mL). The mixture was stirred at room temperature. After completion, the reaction mixture was diluted with ethyl acetate, and washed with saturated sodium bicarbonate aq. and brine. The organic layer was dried over Na_2_SO_4_, filtered, and concentrated under reduced pressure. The conversion was determined by ^1^H NMR spectra of the crude product. The *ee* of the product was determined by HPLC analysis of the epoxides.

### 4.4. Synthesis

5-(*tert*-Butyl)-2-hydroxybenzaldehyde (**2**) [21]. Paraformaldehyde (514.8 mg, 17.2 mmol, 3.0 eq.) was added to a suspension of 4-(*tert*-butyl)phenol (858.9 mg, 5.72 mmol, 1.0 eq.), Et_3_N (4.0 mL, 28.6 mmol, 5.0 eq.) and MgCl_2_ (1635.0 mg, 17.2 mmol, 3.0 eq.) in MeCN (8.5 mL) at 65 °C. The mixture was stirred for 1.5 h. The resulting reaction mixture was cooled to room temperature and quenched with 1 M HCl aq. After the addition of ethyl acetate, the organic layer was separated and the aqueous layer was further extracted with ethyl acetate. The combined organics were washed with brine (×3), dried over Na_2_SO_4_, filtered, and concentrated under reduced pressure. The residue was purified by silica gel column chromatography (hexane/EtOAc = 15:1) to give the desired product **2** (609.4 mg, 60%). Pale yellow oil; *R*_f_ 0.41 (hexane/EtOAc = 15:1); ^1^H NMR (400 MHz, CDCl_3_): δ 10.86 (s, 1H), 9.89 (s, 1H), 7.59 (dd, *J* = 8.8, 2.0 Hz, 1H), 7.51 (d, *J* = 2.8 Hz, 1H), 6.94 (d, *J* = 8.8 Hz, 1H), 1.33 (s, 9H).

5-(*tert*-Butyl)-2-hydroxy-3-(perfluorobutyl)benzaldehyde (**3a**) [19]. Perfluorobutyl iodide (861 µL, 5.0 mmol, 5.0 eq.) and V-70L (17.8 mg, 0.058 mmol, 6 mol%) were added to a suspension of salicylaldehyde **2** (178.2 mg, 1.0 mmol, 1.0 eq.) and cesium carbonate (1310.5 mg, 4.0 mmol, 4.0 eq.) in DMF (1 mL). The mixture was stirred for 30 min at 80 °C in oil bath. The mixture was quenched with 1 M HCl aq. After the addition of Et_2_O, the organic phase was separated and the aqueous phase was extracted with Et_2_O (×2). The combined organic phase was washed with brine (×3), dried over Na_2_SO_4_, filtered, and concentrated under reduced pressure. The residue was purified by silica gel column chromatography (hexane/EtOAc = 10:1, 2 times) to give the desired product **3a** (126.8 mg, 32%). Yellow solid; mp 38 °C; *R*_f_ 0.48 (hexane/EtOAc = 10:1); ^1^H NMR (400 MHz, CDCl_3_): δ 11.64 (s, 1H), 9.95 (s, 1H), 7.75 (dd, *J* = 6.0, 2.4 Hz, 2H), 1.36 (s, 9H); ^19^F NMR (376 MHz, CDCl_3_): δ −80.8 (3F), −108.9 (2F), −122.3 (2F), −125.8 (2F).

5-(*tert*-Butyl)-2-hydroxy-3-(perfluorododecyl)benzaldehyde (**3b**). V-70L (21.6 mg, 0.07 mmol, 15 mol%) was added to a suspension of salicylaldehyde **2** (83.1 mg, 0.5 mmol, 1.0 eq.), perfluorododecyl iodide (1042.0 mg, 1.4 mmol, 3.0 eq.), and cesium carbonate (608.0 mg, 1.9 mmol, 4.0 eq.) in DMF (3.8 mL). The mixture was stirred for 2.5 h at 80 °C in oil bath. The mixture was quenched with 1 M HCl aq. After the addition of Et_2_O, the organic phase was separated and the aqueous phase was extracted with Et_2_O (×2). The combined organic phase was washed with brine (×3), dried over Na_2_SO_4_, filtered, and concentrated under reduced pressure. The residue was filtered over a short pad of silica gel (hexane only to hexane/EtOAc = 10:1) and purified by fluorous solid phase extraction (FSPE) using 40% aq. THF (THF/H_2_O = 60:40) as the fluorophobic solvent and THF as the fluorophilic solvent. Concentration of the THF fraction under reduced pressure gave a residue that was contaminated with BHT, which was removed by silica gel column chromatography (hexane/EtOAc = 30:1) to give the desired product **3b** (186.3 mg, 50%). White solid; mp 101–102 °C; *R*_f_ 0.47 (hexane/EtOAc = 10:1); ^1^H NMR (400 MHz, CDCl_3_): δ 11.65 (s, 1H), 9.95 (s, 1H), 7.76 (dd, *J* = 6.4, 2.0 Hz, 2H), 1.36 (s, 9H); ^13^C NMR (101 MHz, CDCl_3_): δ 196.7, 158.7, 142.7, 134.7, 133.92, 133.85, 133.77, 121.1, 118.8–107.5 (m, C_12_F_25_ tag), 34.4, 31.2; ^19^F NMR (376 MHz, CDCl_3_): δ −80.6 (3F), −108.7 (2F), −121.3 to −121.7 (16F), −122.5 (2F), −125.9 (2F); HRMS-DART (*m*/*z*): [M + H]^+^ calcd for C_23_H_14_F_25_O_2_^+^, 797.0589; found, 797.0593.

Fluorous iron(III) salen complex with C_4_F_9_ tag (**1a**) [19]. Under N_2_ atmosphere, (1*R,*2*R*)-(+)-1,2-diphenylethylenediamine (53.5 mg, 0.25 mmol, 0.5 eq.) was added to a suspension of **3a** (197.7 mg, 0.50 mmol, 1.0 eq.) and 3 A MS in dry-EtOH (4 mL). The mixture was stirred at reflux in oil bath for 22 h; 3 A MS was filtered over a pad of Celite^®^. The filtrate was concentrated under reduced pressure. The residue was purified by silica gel column chromatography (hexane/EtOAc = 10:1) to give the ligand as a yellow solid. The ligand was immediately used for the next ligand exchange. Under N_2_ atmosphere, FeCl_3_ (85.1 mg, 0.52 mmol, 1.1 eq.) was added to a solution of ligand in dry-EtOH (4 mL). The resulting mixture was stirred at reflux in oil bath for 1 h. After the addition of EtOAc, the organic layer was washed with brine (×3), dried over Na_2_SO_4_, filtered, and concentrated under reduced pressure. The residue was purified by silica gel column chromatography (hexane/EtOAc = 10:1 to 1:1) to give the fluorous iron(III) salen complex **1a** (138.0 mg, 52%). Reddish brown solid; mp 130 °C; *R*_f_ 0.40 (CHCl_3_/MeOH = 20:1); HRMS-DART (*m*/*z*): [M + H]^+^ calcd for C_44_H_37_ClF_18_FeN_2_O_2_^+^, 1058.1600; found, 1058.1592.

Fluorous iron(III) salen complex with C_12_F_25_ tag (**1b**). Under N_2_ atmosphere, (1*R*,2*R*)-(+)-1,2-diphenylethylenediamine (29.5 mg, 0.14 mmol, 0.6 eq.) was added to a suspension of **3b** (183.6 mg, 0.23 mmol, 1.0 eq.) and 3 A MS in dry-EtOH (4.6 mL). The mixture was stirred at reflux in an oil bath for 30 min; 3 A MS was filtered over a pad of Celite^®^. The filtrate was concentrated under reduced pressure. The residue was purified by silica gel column chromatography (hexane/EtOAc = 10:1) to give the ligand as a yellow solid. Under N_2_ atmosphere, FeCl_3_ (37.1 mg, 0.23 mmol, 1.0 eq.) was added to a solution of ligand in dry-EtOH (4.6 mL). The resulting mixture was stirred at reflux in oil bath for 30 min. The resulting mixture was concentrated under reduced pressure. After the addition of EtOAc, the organic layer was washed with brine (×3), dried over Na_2_SO_4_, filtered, and concentrated under reduced pressure. The residue was purified by silica gel column chromatography (hexane/EtOAc = 10:1 to 1:1) to give the fluorous iron(III) salen complex **1b** (97.6 mg, 46%). Reddish brown solid; mp 196 °C; *R*_f_ 0.24 (CHCl_3_/MeOH = 20:1); HRMS-DART (*m*/*z*): [M + H]^+^ calcd for C_60_H_37_ClF_50_FeN_2_O_2_^+^, 1858.1089; found, 1858.1087.

Ligand of **1b**. Yellow solid; mp 70–72 °C; *R*_f_ 0.38 (hexane/EtOAc = 10:1); ^1^H NMR (400 MHz, CDCl_3_): δ 8.42 (s, 2H), 7.46 (d, *J* = 2.4 Hz, 2H), 7.32 (d, *J* = 2.4 Hz, 2H), 7.24–7.13 (m, 10H), 4.73 (s, 2H), 1.22 (s, 18H); ^13^C NMR (101 MHz, CDCl_3_): δ 166.5, 158.2, 141.2, 138.6, 132.9, 128.7, 128.0, 119.1, 80.3, 34.1, 31.1; ^19^F NMR (376 MHz, CDCl_3_): δ −80.7 (6F), −108.5 (4F), −121.3 to −121.8 (32F), −122.6 (4F), −126.0 (4F); HRMS-DART (*m*/*z*): [M + H]^+^ calcd for C_60_H_39_F_50_N_2_O_2_^+^, 1769.2208; found, 1769.2208.

Jacobsen-type iron(III) salen complex (**1c**) [19]. Under N_2_ atmosphere, (1*R,*2*R*)-(+)-1,2-diphenylethylenediamine (127.2 mg, 0.6 mmol, 0.6 eq.) was added to a suspension of 3,5-di-*tert*-butyl-2-hydroxybenzaldehyde (234.5 mg, 1.0 mmol, 1.0 eq.) and 3 A MS in dry-EtOH (20 mL). The mixture was stirred at reflux in an oil bath for 1 h; 3 A MS was filtered over a pad of Celite^®^. The filtrate was concentrated under reduced pressure. The residue was purified by silica gel column chromatography (hexane/EtOAc = 10:1) to give the ligand as a yellow solid (286.6 mg). Under N_2_ atmosphere, FeCl_3_ (162.6 mg, 1.0 mmol, 1.0 eq.) was added to a solution of ligand in dry-EtOH (20 mL). The resulting mixture was stirred at reflux in oil bath for 1 h. The resulting mixture was concentrated under reduced pressure. After the addition of EtOAc, the organic layer was washed with brine (×3), dried over Na_2_SO_4_, filtered, and concentrated under reduced pressure. The residue was purified by silica gel column chromatography (hexane/EtOAc = 10:1 to 1:1) to give the Jacobsen-type iron(III) salen complex **1c** (242.8 mg, 33%). Reddish brown solid; mp 183–184 °C; *R*_f_ 0.39 (CHCl_3_/MeOH = 20:1); HRMS-DART (*m*/*z*): [M + H]^+^ calcd for C_44_H_55_ClFeN_2_O_2_^+^, 734.3296; found, 734.3296.

Ligand of **1c** [22]. Yellow solid; mp 199–200 °C; *R*_f_ 0.50 (hexane/EtOAc = 10:1); ^1^H NMR (400 MHz, CDCl_3_): δ 8.39 (s, 2H), 7.31–7.30 (m, 2H), 7.22–7.14 (m, 10H), 6.98–6.97 (m, 2H), 4.72 (s, 2H), 1.423–1.418 (m, 18H), 1.224–1.218 (m, 18H).

2,2,3-Triphenyloxirane (**5a**) [23]. Following the general procedure, olefin **4a** was converted to epoxide. The conversion was determined by integrating the signals of the olefin (6.97 ppm) and the epoxide (4.33 ppm). The crude product was subsequently purified through silica gel column chromatography (hexane/EtOAc = 20:1), resulting in the isolation of epoxide **5a** (95% yield). White solid; mp 70–72 °C; R_f_ 0.43 (hexane/EtOAc = 20:1); ^1^H NMR (400 MHz, CDCl_3_): δ 7.39–7.29 (m, 5H), 7.20 (s, 5H), 7.15–7.13 (m, 3H), 7.05–7.02 (m, 2H), 4.33 (s, 1H).

(*E*)-1-Methyl-4-styrylbenzene (**4e**) [24]. Under N_2_ atmosphere, diethyl benzylphosphonate (1.6 mL, 7.5 mmol, 5.0 eq.) in dry-Et_2_O (3.0 mL) was added to a suspension of sodium *tert*-butoxide (721.5 mg, 7.5 mmol, 5.0 eq.) in dry-Et_2_O (5.0 mL). The reaction mixture was stirred at room temperature for 15 min. 4-Methylbenzaldehyde (177 µL, 1.5 mmol, 1.0 eq.) was added to the reaction mixture, and stirred for 30 min. The mixture was quenched with 1 M HCl aq. The reaction mixture was diluted with EtOAc, the organic phase was separated and the aqueous phase was extracted with EtOAc. The combined organic phase was washed with brine (×3), dried over Na_2_SO_4_, filtered, and concentrated under reduced pressure. The residue was purified by silica gel column chromatography (hexane/EtOAc = 30:1) to give the desired product **4e** (231.1 mg, 79%). White solid; mp 114 °C; *R*_f_ 0.59 (hexane/EtOAc = 10:1); ^1^H NMR (400 MHz, CDCl_3_): δ 7.52–7.49 (m, 2H), 7.42 (d, *J* = 8.4 Hz, 2H), 7.35 (t, *J* = 7.2 Hz, 2H), 7.26–7.22 (m, 1H), 7.17 (d, *J* = 7.6 Hz, 2H), 7.08 (d, *J* = 2.4 Hz, 2H), 2.36 (s, 3H).

(*E*)-1-Methoxy-4-styrylbenzene (**4f**) [24]. Diethyl benzylphosphonate (1.1 mL, 5.5 mmol, 5.0 eq.) in dry-Et_2_O (3.0 mL) was added to a suspension of sodium *tert*-butoxide (528.0 mg, 5.5 mmol, 5.0 eq.) in dry-Et_2_O (3.0 mL). The reaction mixture was stirred at 50 °C for 10 min. 4-Methoxybenzaldehyde (133 µL, 1.1 mmol, 1.0 eq.) was added to the reaction mixture, and stirred for 30 min. The mixture was quenched with 1 M HCl aq. The reaction mixture was diluted with Et_2_O, the organic phase was separated and the aqueous phase was extracted with Et_2_O. The combined organic phase was washed with brine (×3), dried over Na_2_SO_4_, filtered, and concentrated under reduced pressure. The residue was purified by silica gel column chromatography (hexane only) to give the desired product **4f** (178.1 mg, 77%). White solid; mp 130 °C; *R*_f_ 0.31 (hexane/EtOAc = 20:1); ^1^H NMR (400 MHz, CDCl_3_): δ 7.50–7.44 (m, 4H), 7.34 (t, *J* = 7.2 Hz, 2H), 7.26–7.21 (m, 2H), 7.07 (d, *J* = 16.4 Hz, 1H), 6.97 (d, *J* = 16.4 Hz, 1H), 6.90 (dt, *J* = 8.8, 3.2 Hz, 2H), 3.83 (s, 3H).

(*E*)-1-Fluoro-4-styrylbenzene (**4g**) [25]. Diethyl benzylphosphonate (1.6 mL, 7.5 mmol, 5.0 eq.) in dry-Et_2_O (3.0 mL) was added to a suspension of sodium *tert*-butoxide (723.0 mg, 7.5 mmol, 5.0 eq.) in dry-Et_2_O (5.0 mL). The reaction mixture was stirred at room temperature for 30 min. 4-Fluorobenzaldehyde (158 µL, 1.5 mmol, 1.0 eq.) was added to the reaction mixture, and stirred for 30 min. The mixture was quenched with 1 M HCl aq. The reaction mixture was diluted with EtOAc, the organic phase was separated and the aqueous phase was extracted with EtOAc. The combined organic phase was washed with brine (×3), dried over Na_2_SO_4_, filtered, and concentrated under reduced pressure. The residue was purified by silica gel column chromatography (hexane/EtOAc = 10:1) to give the desired product **4g** (239.8 mg, 81%). White solid; mp 94–95 °C; *R*_f_ 0.78 (hexane/EtOAc = 10:1); ^1^H NMR (400 MHz, CDCl_3_): δ 7.50–7.46 (m, 4H), 7.36 (t, *J* = 7.6 Hz, 2H), 7.28–7.24 (m, 1H), 7.12–7.00 (m, 4H); ^19^F NMR (376 MHz, CDCl_3_): δ −114.2 (1F).

(*E*)-2-Styrylnaphthalene (**4h**) [26]. Diethyl benzylphosphonate (1.6 mL, 7.5 mmol, 5.0 eq.) in dry-Et_2_O (3.0 mL) was added to a suspension of sodium *tert*-butoxide (724.7 mg, 7.5 mmol, 5.0 eq.) in dry-Et_2_O (5.0 mL). The reaction mixture was stirred at room temperature for 15 min. 2-Naphthaldehyde (234.6 mg, 1.5 mmol, 1.0 eq.) was added to the reaction mixture, and stirred for 30 min. The mixture was quenched with 1 M HCl aq. The reaction mixture was diluted with EtOAc, the organic phase was separated and the aqueous phase was extracted with EtOAc. The combined organic phase was washed with brine (×3), dried over Na_2_SO_4_, filtered, and concentrated under reduced pressure. The residue was purified by silica gel column chromatography (hexane EtOAc = 30:1) to give the desired product **4h** (228.0 mg, 66%). White solid; mp 147 °C; *R*_f_ 0.66 (hexane/EtOAc = 30:1); ^1^H NMR (400 MHz, CDCl_3_): δ 7.86–7.81 (m, 4H), 7.75 (d, *J* = 8.8 Hz, 1H), 7.57 (d, *J* = 8.4 Hz, 2H), 7.50–7.43 (m, 2H), 7.39 (t, *J* = 8.0 Hz, 2H), 7.31–7.22 (m, 3H).

3-(Methoxymethoxy)-3,7-dimethylocta-1,6-diene (**4i**). Under N_2_ atmosphere, MOMCl (456 µL, 6.0 mmol, 2.0 eq.) was added to a solution of linalool (467.2 mg, 3.0 mmol, 1.0 eq.), DIPEA (1.55 mL, 9.0 mmol, 3.0 eq.), and DMAP (10.3 mg, 0.084 mmol, 2.7 mol%) in dry-DCM (10 mL). The mixture was stirred at room temperature. After completion, the reaction mixture was quenched with 10% citric acid aq. After the addition of DCM, the organic layer was separated, and the aqueous layer was further extracted with DCM. The combined organics were dried over Na_2_SO_4_, filtered, and concentrated under reduced pressure. The residue was purified by silica gel column chromatography (hexane/EtOAc = 2:1) to give the desired product **4i** (540.9 mg, 91%). Colorless oil; *R*_f_ 0.73 (hexane/EtOAc = 5:1); ^1^H NMR (400 MHz, CDCl_3_): δ 5.87–5.80 (m, 1H), 5.181–5.178 (m, 1H), 5.15–5.14 (m, 1H), 5.12–5.08 (m, 1H), 4.69 (d, *J* = 6.8 Hz, 1H), 4.63 (d, *J* = 7.2 Hz, 1H), 3.37 (s, 3H), 2.04–1.98 (m, 2H), 1.68 (br s, 3H), 1.60 (br s, 3H), 1.59–1.55 (m, 2H), 1.32 (s, 3H); ^13^C NMR (101 MHz, CDCl_3_): δ 143.0, 131.6, 124.6, 114.6, 91.8, 78.7, 55.4, 41.1, 25.8, 23.1, 22.6, 17.8; HRMS-DART (*m*/*z*): [M + H]^+^ calcd for C_12_H_23_O_2_^+^, 199.1693; found, 199.1692.

*trans*-Stilbene epoxide (**5b**) [27]. Following the general procedure, olefin **4b** was converted to epoxide. The conversion was calculated using the integral of the olefin signal (7.11 ppm) and the epoxide signal (3.86 ppm); conv. 100%. The obtained crude product was purified by silica gel column chromatography (hexane/EtOAc = 20:1) to give the epoxide **5b**. White solid; mp 66 °C; *R*_f_ 0.51 (hexane/EtOAc = 20:1); ^1^H NMR (400 MHz, CDCl_3_): δ 7.40–7.30 (m, 10H), 3.86 (s, 2H).

*trans*-β-Methylstyrene epoxide (**5c**) [28]. Following the general procedure, *cis*-type olefin **4c** was converted to epoxide. The conversion was calculated using the integral of the olefin signal (5.79 ppm) and the epoxide signal (3.04 ppm); *trans*-type epoxide only, conv. 100%. The obtained crude product was purified by silica gel column chromatography (hexane/EtOAc = 20:1) to give the epoxide **5c**. Colorless oil; *R*_f_ 0.33 (hexane/EtOAc = 20:1); ^1^H NMR (400 MHz, CDCl_3_): δ 7.36–7.25 (m, 5H), 3.57 (d, *J* = 2.0 Hz, 1H), 3.04 (dq, *J* = 5.2, 2.4 Hz, 1H), 1.45 (d, *J* = 5.2 Hz, 3H).

α-Methylstyrene epoxide (**5d**) [27]. Following the general procedure, olefin **4d** was converted to epoxide. The conversion was calculated using the integral of the olefin signal (5.28 ppm) and the epoxide signal (2.80 ppm); conv. 100%. The obtained crude product was purified by silica gel column chromatography (hexane/EtOAc = 20:1) to give the epoxide **5d**. Colorless oil; *R*_f_ 0.26 (hexane/EtOAc = 20:1); ^1^H NMR (400 MHz, CDCl_3_): δ 7.39–7.25 (m, 5H), 2.98 (d, *J* = 5.2 Hz, 1H), 2.80 (dd, *J* = 5.6, 0.8 Hz, 1H), 1.72 (s, 3H).

2-Phenyl-3-(*p*-tolyl)oxirane (**5e**) [28]. Following the general procedure, olefin **4e** was converted to epoxide. The conversion was calculated using the integral of the olefin signal (7.07 ppm) and the epoxide signal (3.83 ppm); conv. 100%. The obtained crude product was purified by silica gel column chromatography (hexane/EtOAc = 20:1) to give the epoxide **5e**. White solid; mp 56 °C; *R*_f_ 0.35 (hexane/EtOAc = 20:1); ^1^H NMR (400 MHz, CDCl_3_): δ 7.40–7.31 (m, 5H), 7.26–7.23 (m, 2H), 7.19 (d, *J* = 8.0 Hz, 2H), 3.86 (d, *J* = 2.0 Hz, 1H), 3.83 (d, *J* = 1.6 Hz, 1H), 2.37 (s, 3H).

2-(4-Methoxyphenyl)-3-phenyloxirane (**5f**) [27]. Following the general procedure, olefin **4f** was converted to epoxide. The conversion was calculated using the integral of the olefin signal (7.07 ppm) and the epoxide signal (3.85 ppm); conv. 100%. The obtained crude product was purified by silica gel column chromatography (hexane/EtOAc = 10:1) to give the epoxide **5f**. White solid; mp 77–78 °C; *R*_f_ 0.37 (hexane/EtOAc = 10:1); ^1^H NMR (400 MHz, CDCl_3_): δ 7.39–7.24 (m, 7H), 6.91 (dt, *J* = 2.0, 8.8 Hz, 2H), 3.85 (d, *J* = 2.0 Hz, 1H), 3.81 (m, 4H).

2-(4-Fluorophenyl)-3-phenyloxirane (**5g**) [28]. Following the general procedure, olefin **4g** was converted to epoxide. The conversion was evaluated using the substrate-derived signal (7.51–7.46 ppm) and the epoxide signal (3.83 ppm); conv. 100%. The obtained crude product was purified by silica gel column chromatography (hexane/EtOAc = 30:1) to give the epoxide **5g**. White solid; mp 73 °C; *R*_f_ 0.38 (hexane/EtOAc = 20:1); ^1^H NMR (400 MHz, CDCl_3_): δ 7.41–7.29 (m, 7H), 7.10–7.04 (m, 2H), 3.85 (d, *J* = 1.6 Hz, 1H), 3.83 (d, *J* = 1.6 Hz, 1H); ^19^F NMR (376 MHz, CDCl_3_): δ −113.5 (1F).

2-(Naphthalen-2-yl)-3-phenyloxirane (**5h**) [29]. Following the general procedure, olefin **4h** was converted to epoxide. The conversion was calculated using the substrate-derived signal (7.57 ppm) and the epoxide signal (3.97 ppm); conv. 52%. The obtained crude product was purified by silica gel column chromatography (hexane/EtOAc = 30:1) to give the epoxide **5h**. White solid; mp 118–119 °C; *R*_f_ 0.34 (hexane/EtOAc = 20:1); ^1^H NMR (400 MHz, CDCl_3_): δ 7.88–7.83 (m, 4H), 7.53–7.46 (m, 2H), 7.44–7.33 (m, 6H), 4.04 (d, *J* = 1.6 Hz, 1H), 3.97 (d, *J* = 1.6 Hz, 1H).

3-(3-(Methoxymethoxy)-3-methylpent-4-en-1-yl)-2,2-dimethyloxirane (**5i**). Following the general procedure, olefin **4i** was converted to epoxide. The conversion was calculated using the integral of the olefin signal (5.12–5.08 ppm) and the epoxide signal (2.72 ppm); monoxide only, conv. 100%. The obtained crude product was purified by silica gel column chromatography (hexane/EtOAc = 5:1) to give the epoxide **5i**. Colorless oil; *R*_f_ 0.38 (hexane/EtOAc = 5:1); ^1^H NMR (400 MHz, CDCl_3_): δ 5.86–5.78 (m, 1H), 5.20–5.15 (m, 2H), 4.71–4.68 (m, 1H), 4.62 (d, *J* = 6.8 Hz, 1H), 3.37 (s, 3H), 2.72 (t, *J* = 5.6 Hz, 1H), 1.80–1.53 (m, 5H), 1.32 (d, *J* = 2.8 Hz, 3H), 1.31 (s, 3H), 1.27 (d, *J* = 2.8 Hz, 3H); ^13^C NMR (101 MHz, CDCl_3_): δ 142.7, 142.5, 115.1, 115.0, 91.8, 78.3, 64.6, 58.5, 55.4, 37.72, 37.69, 25.0, 23.67, 23.64, 23.1, 23.0, 18.78, 18.76; HRMS-DART (*m*/*z*): [M + H]^+^ calcd for C_12_H_23_O_3_^+^, 215.1642; found, 215.1642.

## Figures and Tables

**Table 1:** Air-epoxidation reaction with fluorous iron(III) salen complexes **1a**, **1b**, and **1c**.

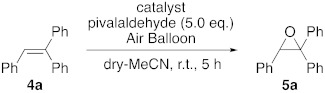		
Entry	Catalyst (mol%)	Conv. (%) ^a^
1	**1a** (1)	73
2	**1b** (1)	100 (98) ^b^
3	**1b** (0.5)	100 (95) ^b^
4	**1b** (0.1)	6
5	**1c** (0.5)	53
6	-	5

^a^ Determined by ^1^H NMR. ^b^ Isolated yield.

**Table 2:** Optimization of co-oxidant aldehydes.

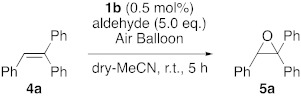							
Entry	Aldehyde	Time (h)	Conv. (%) ^a^	Entry	Aldehyde	Time (h)	Conv. (%) ^a^
1	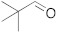	5	100	7	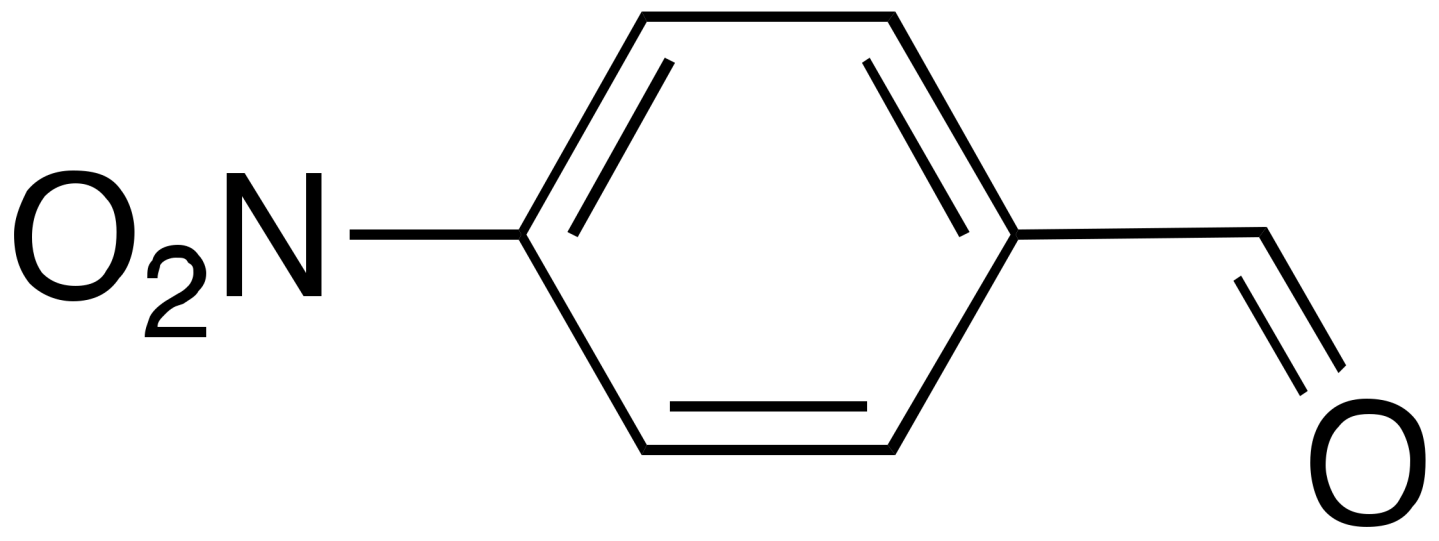	24	n.r. ^b^
2	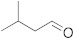	24	82	8	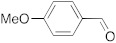	24	trace
3	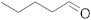	24	10	9	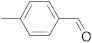	24	n.r. ^b^
4	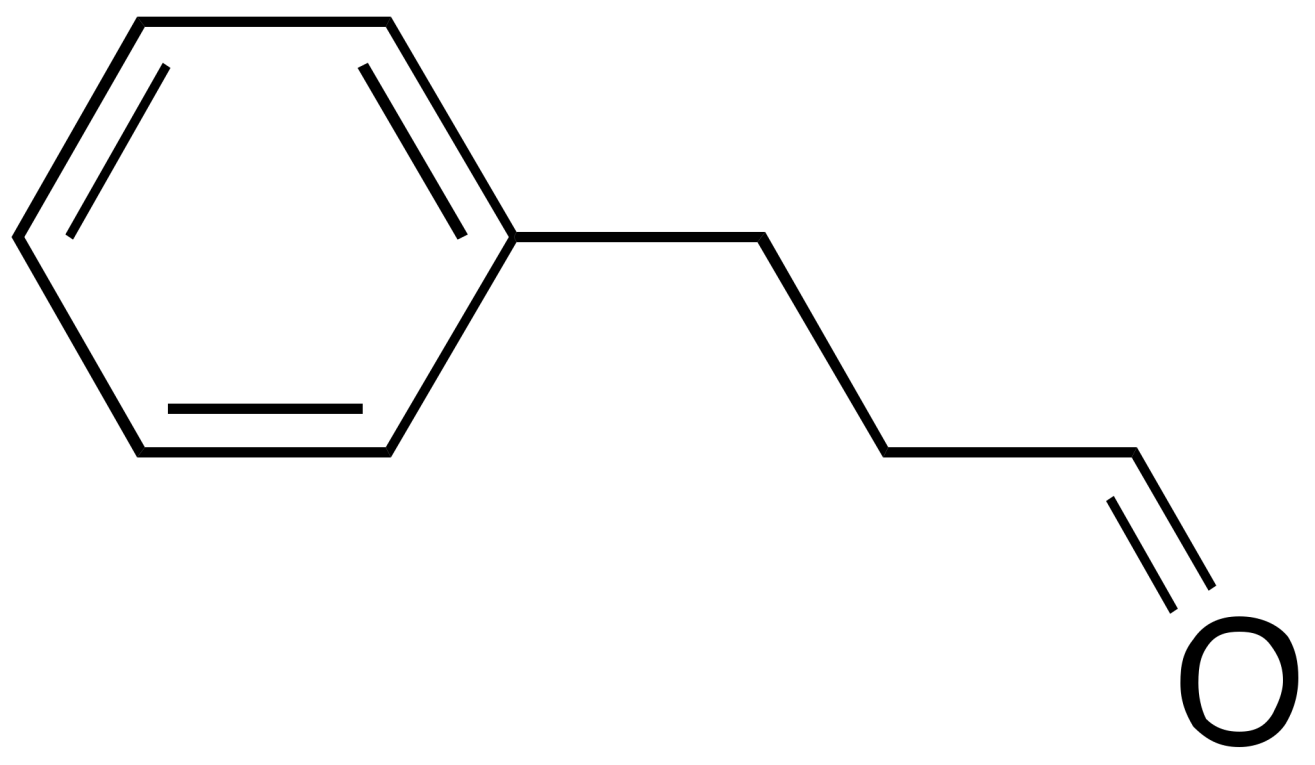	24	trace	10	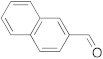	24	n.r. ^b^
5	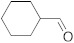	24	trace	11	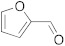	24	trace
6	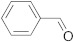	24	n.r. ^b^	12	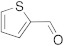	24	trace

^a^ Determined by ^1^H NMR. ^b^ “n.r.” means “no reaction”.

**Table 3:** Solvent effects in air-epoxidation reactions.

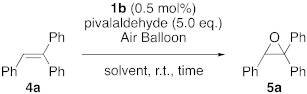			
Entry	Solvent	Time (h)	Conv. (%) ^a^
1	dry-MeCN	5	100
2	*^t^*BuOH	8	100
3	dry-MeOH	24	trace
4	dry-DMF	24	trace
5	dry-THF	24	trace
6	dry-DCM	24	96
7	2,3,4,5,6-pentafluorotoluene	24	98

^a^ Determined by ^1^H NMR.

**Table 4:** Air-epoxidation reactions using various substrates.

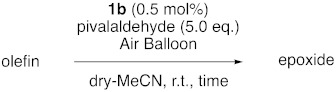									
Entry	Olefin	Epoxide	Time (h)	Conv. (%) ^a^	Entry	Olefin	Epoxide	Time (h)	Conv. (%) ^a^
1	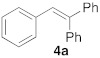	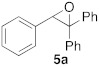	5	100	6	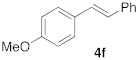	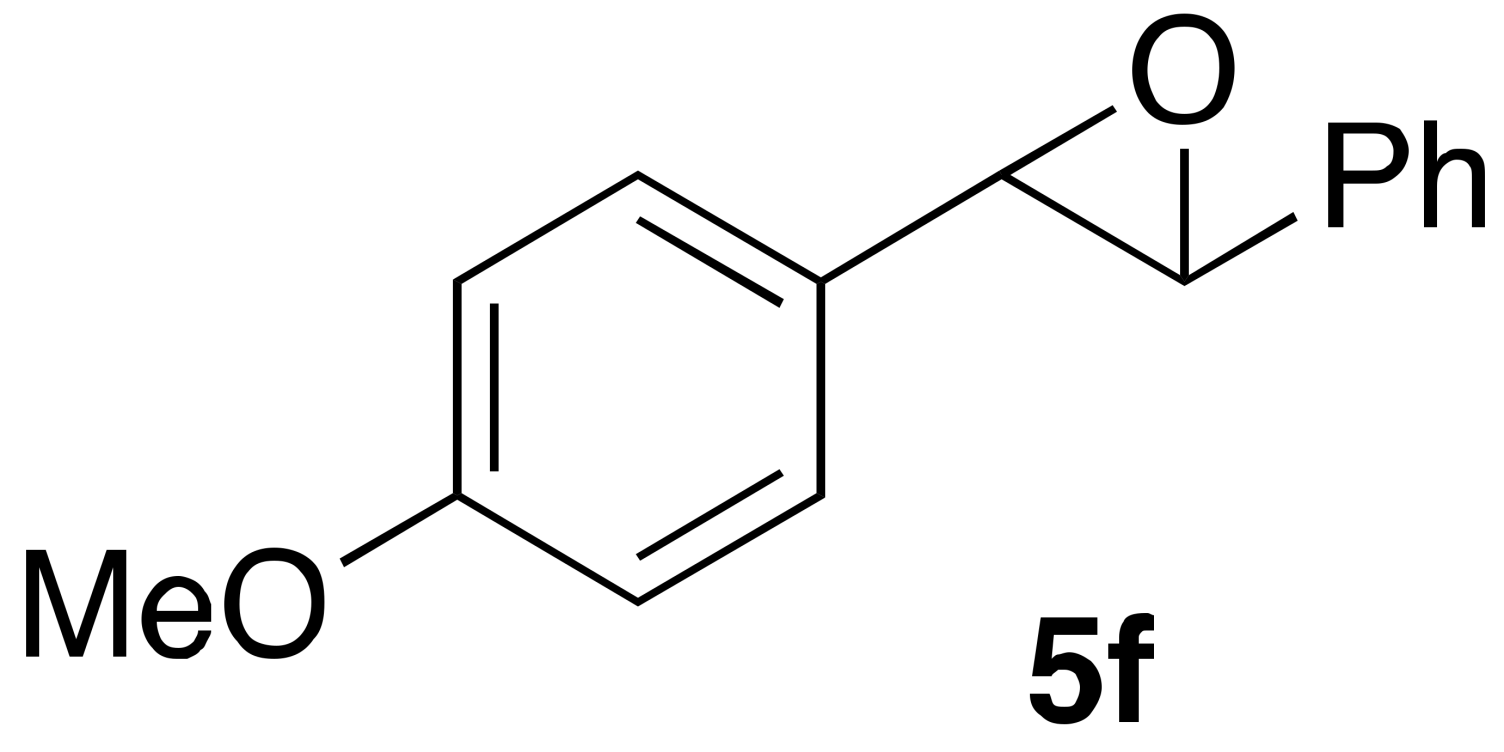	5	100
2	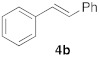	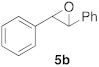	7	100	7	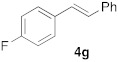	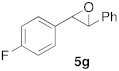	5	100
3	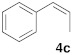	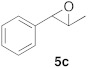	6	100	8	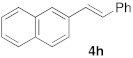	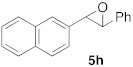	24	52
4	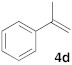	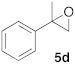	7	100	9 ^b^	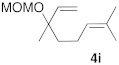	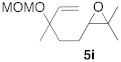	5	100 (5**i**:5**i**’ = 100:0)
5	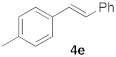	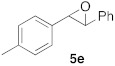	6	100			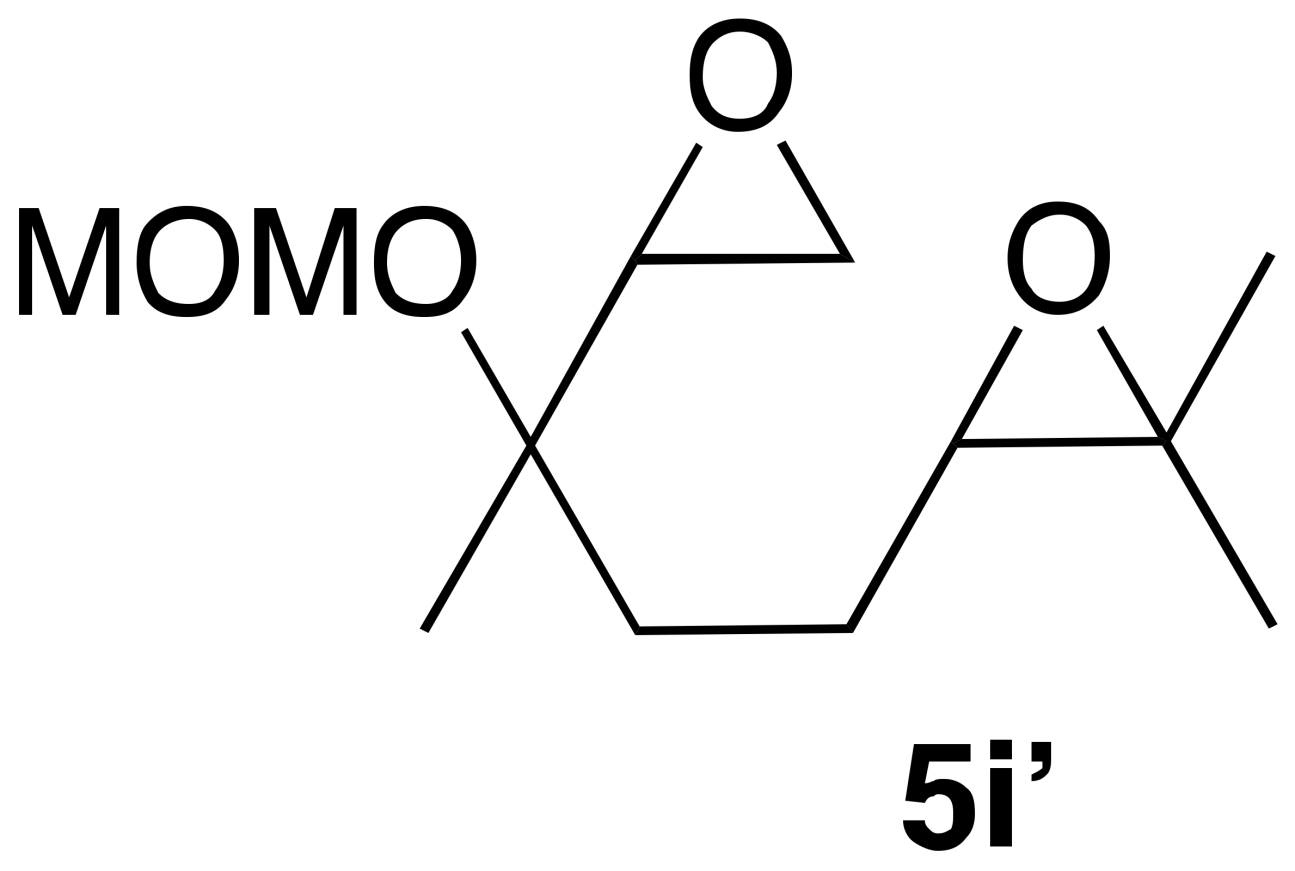		

^a^ Determined by ^1^H NMR. ^b^ “MOM” represents the “methoxymethyl group”.

## Data Availability

All data from this study are reported in the text or in the Supplementary Materials.

## Supplementary Materials

The following supporting information can be downloaded at: https://www.mdpi.com/1420-3049/29/5/966/s1, Copies of ^1^H NMR, ^13^C NMR, ^19^F NMR spectra of compounds (ligand of **1a**–**c**, **3a**, **3b**, **5a**–**i**) can be found in the Supplementary Materials.

## Appendix A

The pressure inside the balloon was crucial for the successful progression of the reaction. A double-layered rubber balloon (9 inches, Sansyo Co., Ltd., Tokyo, Japan) was filled with air, and connected to a 10 mL eggplant flask using a three-way valve. The dimensions of the inflated balloons are as follows: width = 7.5 inches, height = 8.3 inches.
