# Supplementary material for: Practical Epoxidation of Olefins Using Air and Ubiquitous Iron-Based Fluorous Salen Complex
Source: Molecules. 2024 Feb 22;29(5):966. doi: 10.3390/molecules29050966 (PMC11154283; doi:10.3390/molecules29050966)

Practical Epoxidation of Olefins using Air and Ubiquitous Iron-based Fluorous Salen Complex

Yamato Kato, Miho Kanoh, Hina Kobayashi, Takayuki Shioiri, and Masato Matsugi*

Faculty of Agriculture, Meijo University, 1-501 Shiogamaguchi, Tempaku, Nagoya 468-8502, Japan.

Table of contents

1. HPLC chromatograms of epoxidation reaction S2, S3
2. ^{1}H NMR, ^{13}C NMR, ^{19}F NMR spectra S4–S32

1. HPLC chromatograms of epoxidation reaction

HPLC analysis of Air-epoxidation reaction with C_4F_9 Fe(III) salen complex

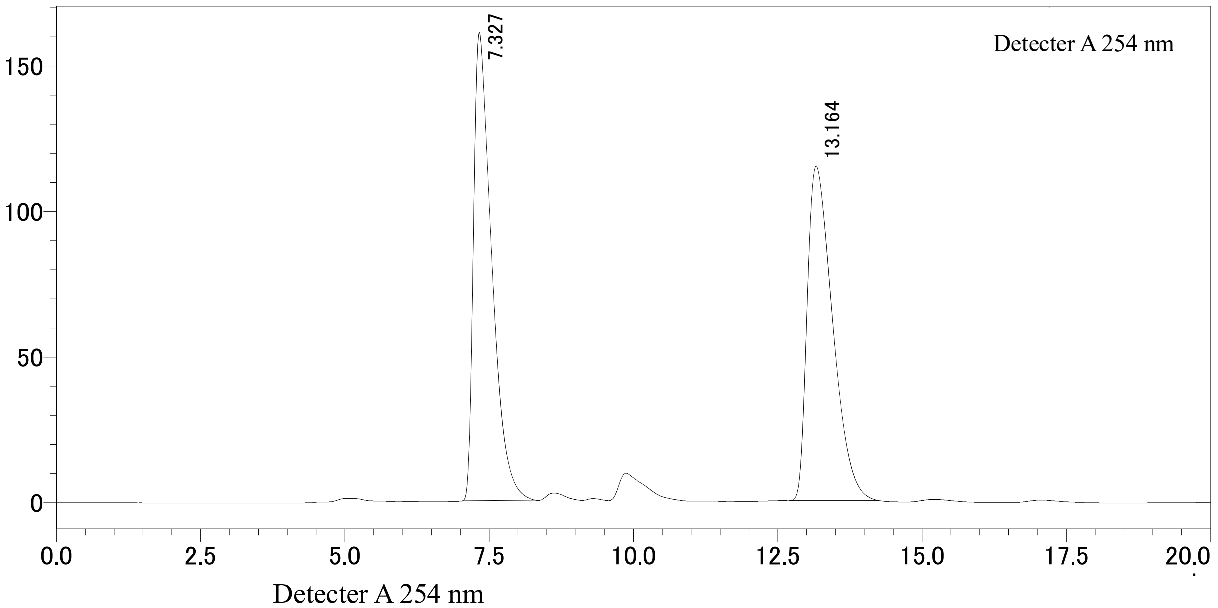

Detecter A 254 nm

Peak	Retention Time	Area	Height	Concentration
1	7.327	3536450	160801	50.076
2	13.164	3525655	114927	49.924
Sum		7062105	275728	

The *ee* of product was determined by HPLC. [254 nm, DAICEL CHIRALCEL OD-3 column, hexane/*i*-PrOH = 9:1, flow rate 0.4 mL/min, t_R = 7.33 min, t_R = 13.16 min]

HPLC analysis of Air-epoxidation reaction with $C_{12}F_{25}$ Fe(III) salen complex

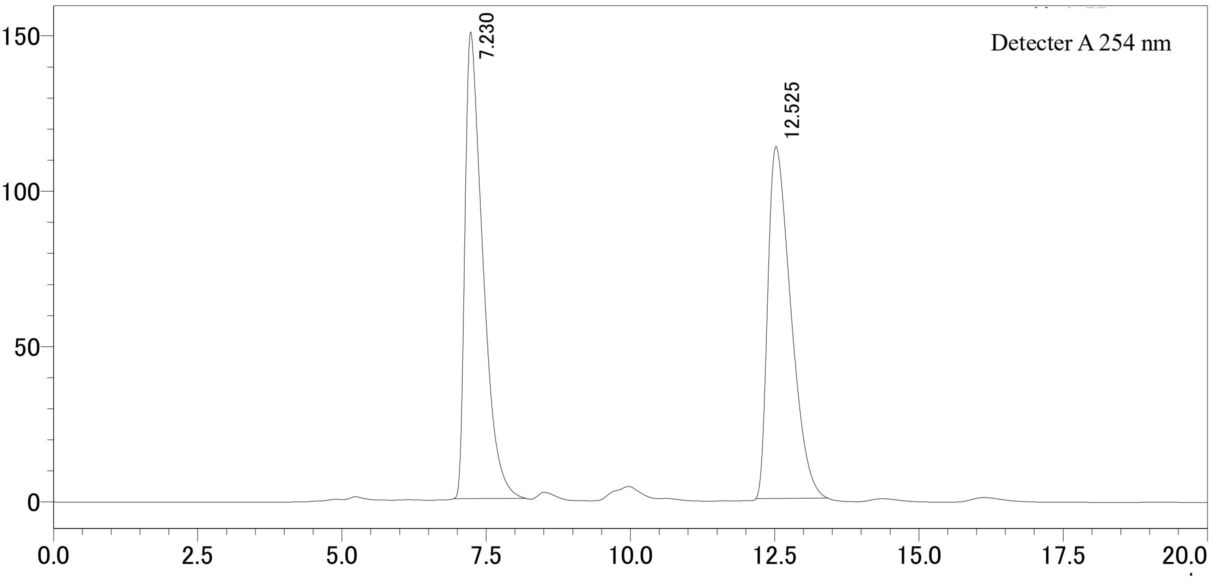

Detecter A 254 nm

Peak	Retention Time	Area	Height	Concentration
1	7.230	3224885	150135	50.583
2	12.525	3150610	113220	49.417
Sum		6375495	263355	

The *ee* of product was determined by HPLC. [254 nm, DAICEL CHIRALCEL OD-3 column, hexane/*i*-PrOH = 9:1, flow rate 0.4 mL/min, t_R = 7.23 min, t_R = 12.53 min]

HPLC analysis of racemic compound

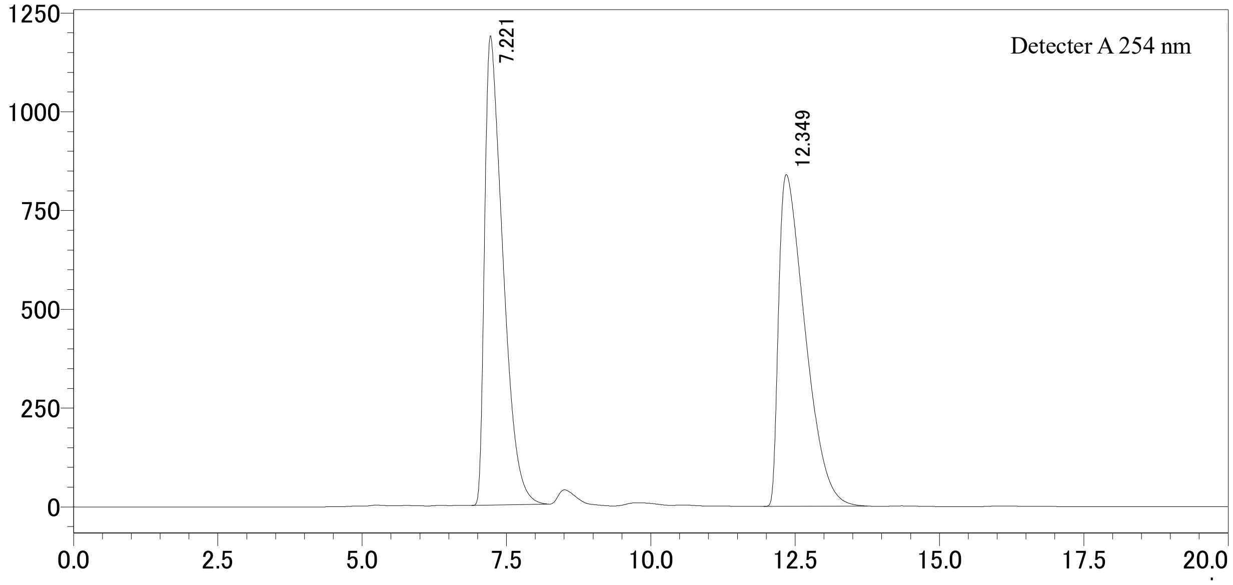

Detecter A 254 nm

Peak	Retention Time	Area	Height	Concentration
1	7.221	26125258	1187330	49.401
2	12.349	26758401	839992	50.599
Sum		52883659	2027322	

The *ee* of product was determined by HPLC. [254 nm, DAICEL CHIRALCEL OD-3 column, hexane/*i*-PrOH = 9:1, flow rate 0.4 mL/min, t_R = 7.22 min, t_R = 12.35 min]

2. ^{1}H NMR, ^{13}C NMR, ^{19}F NMR spectra

(**2**) ^{1}H NMR, 400 MHz, $CDCl_3$

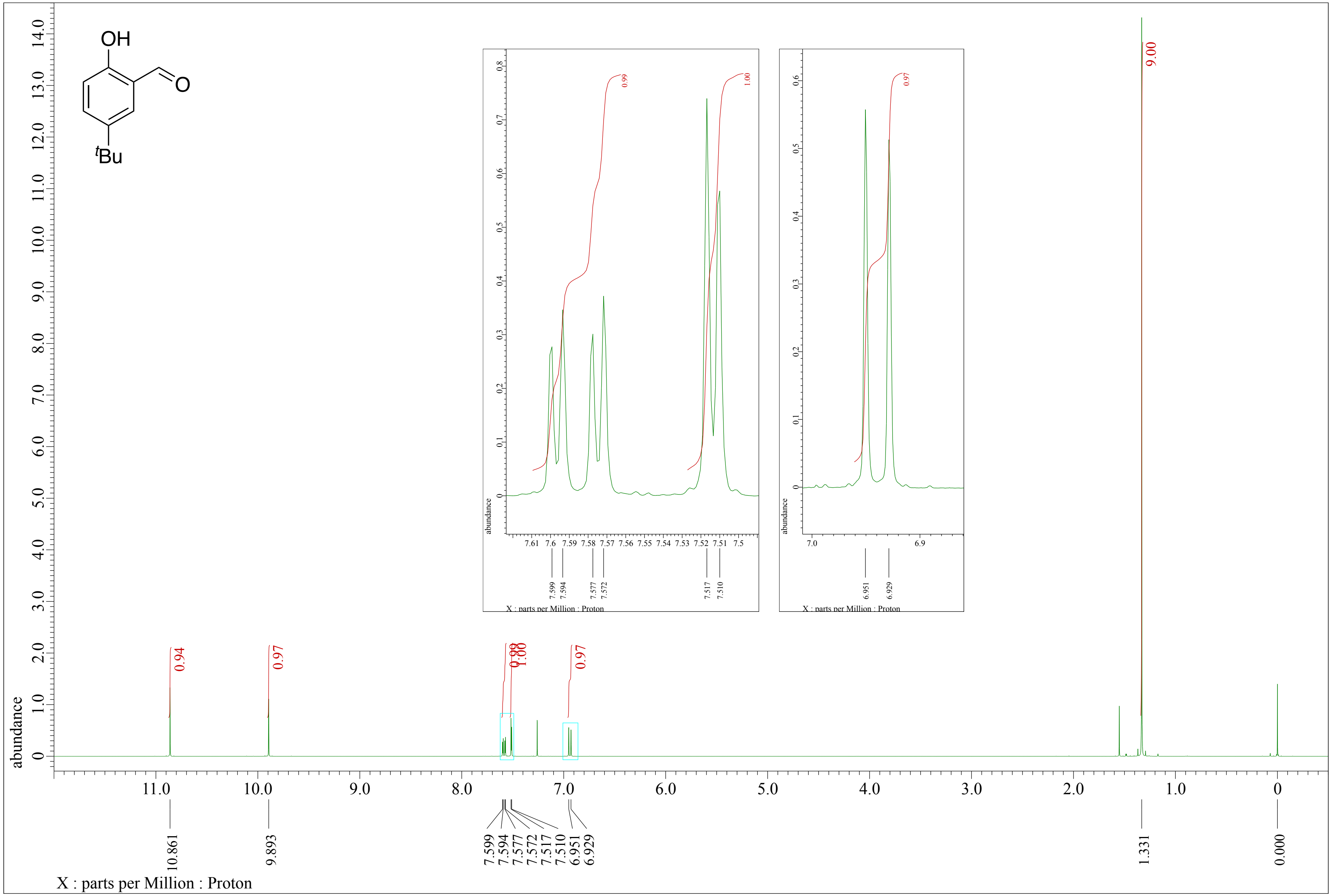

(**3a**) ^{1}H NMR, 400 MHz, $CDCl_3$

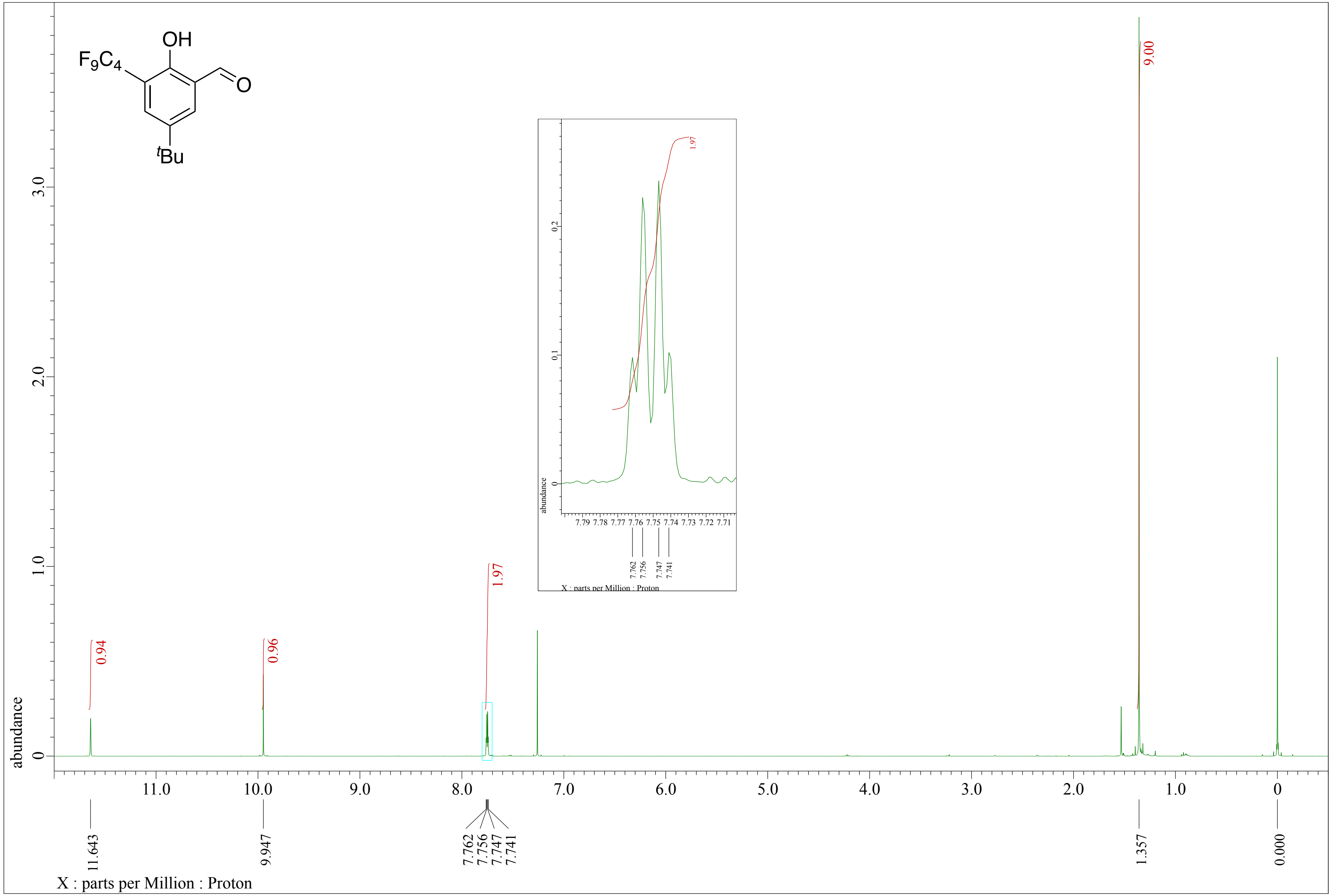

(**3a**) ^{19}F NMR, 376 MHz, $CDCl_3$

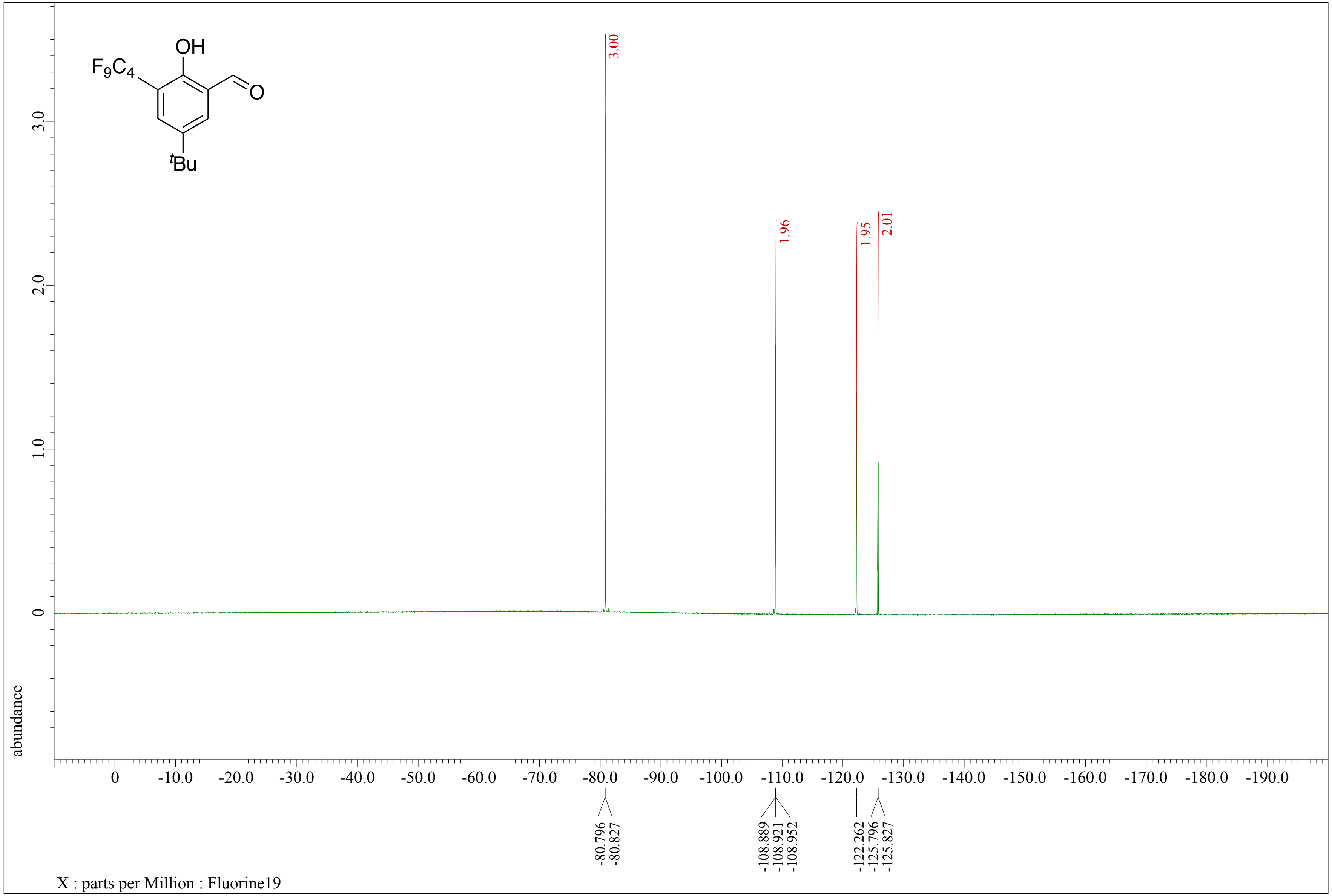

(**3b**) ^{1}H NMR, 400 MHz, $CDCl_3$

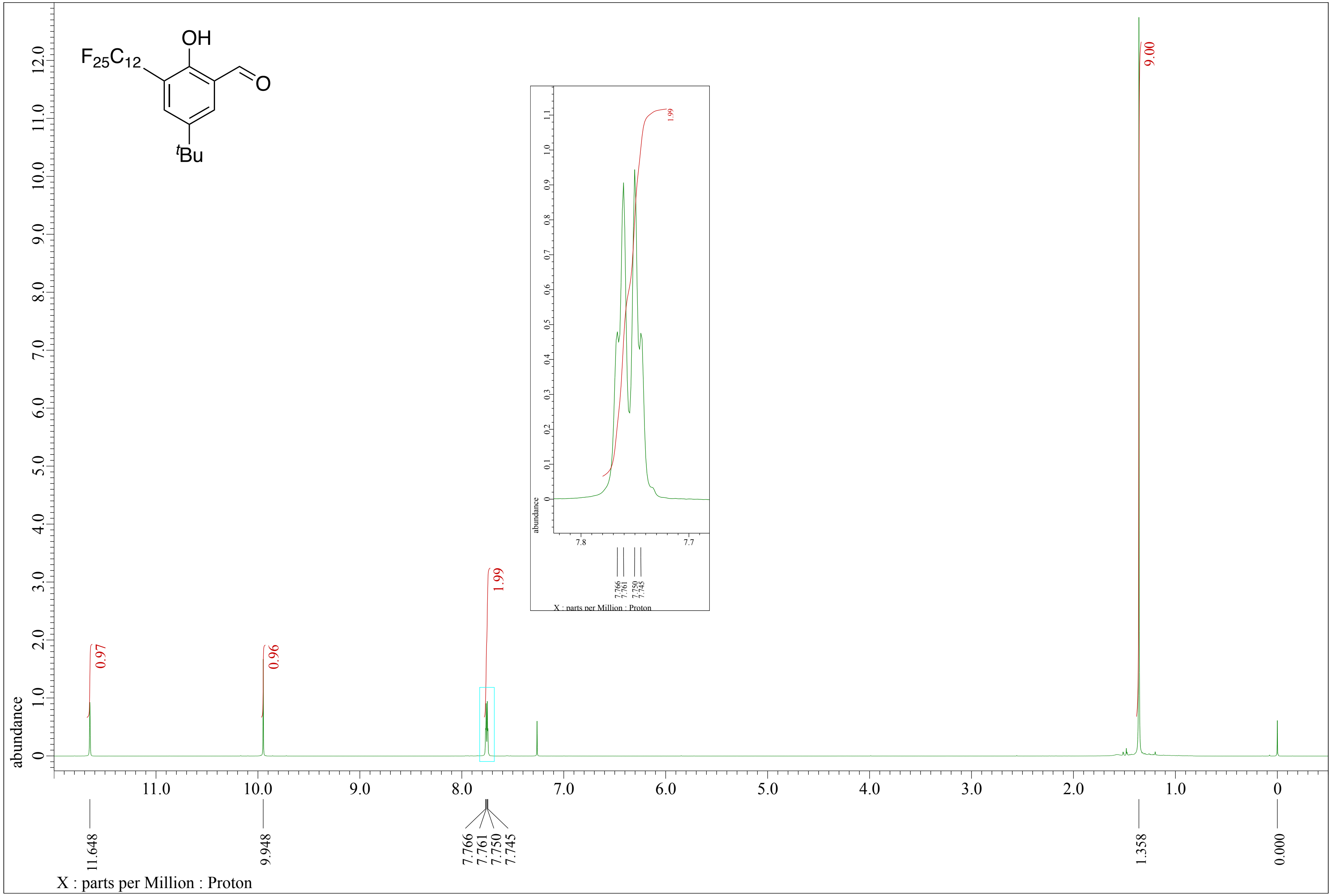

(**3b**) ^{13}C NMR, 101 MHz, $CDCl_3$

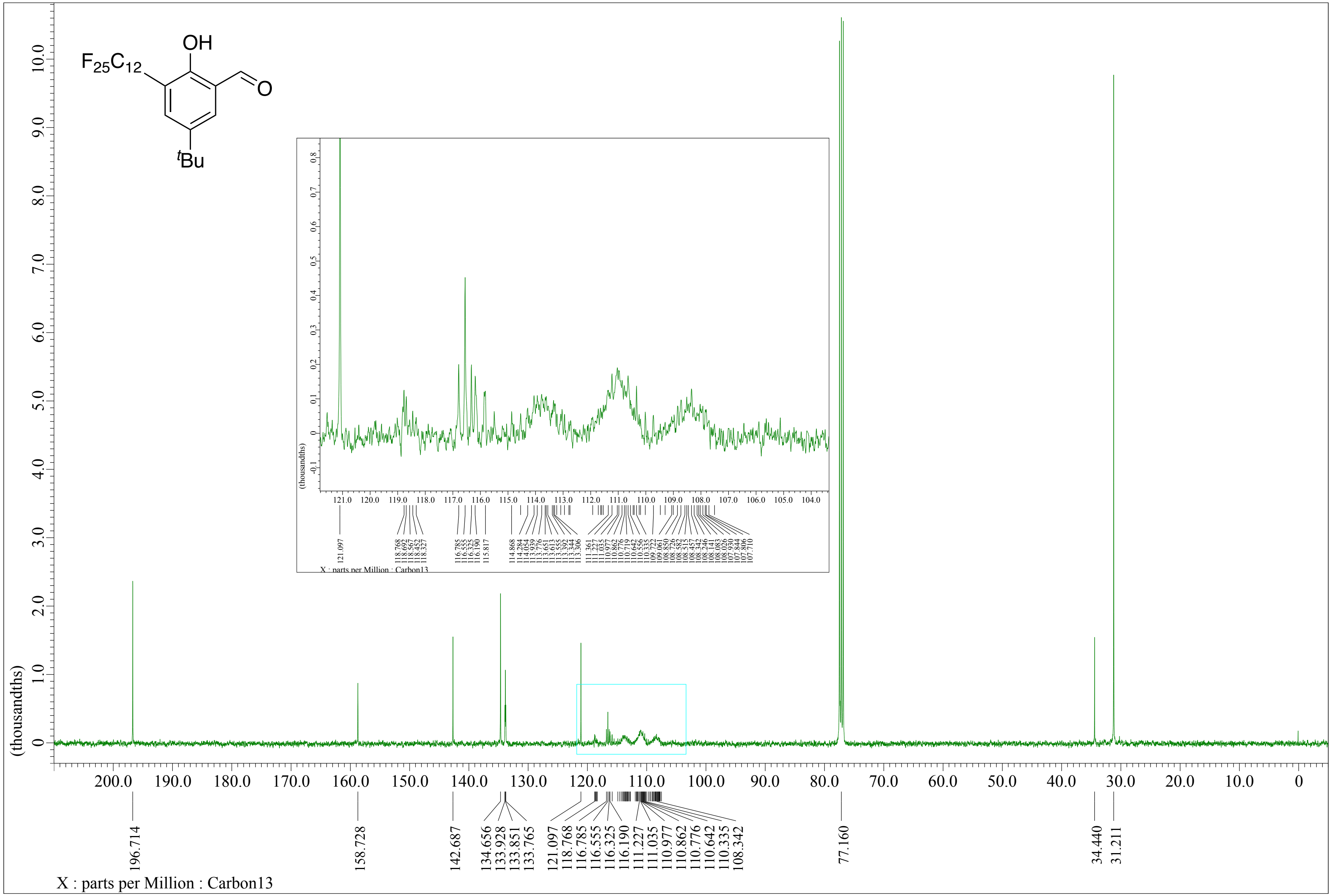

(**3b**) ^{19}F NMR, 376 MHz, $CDCl_3$

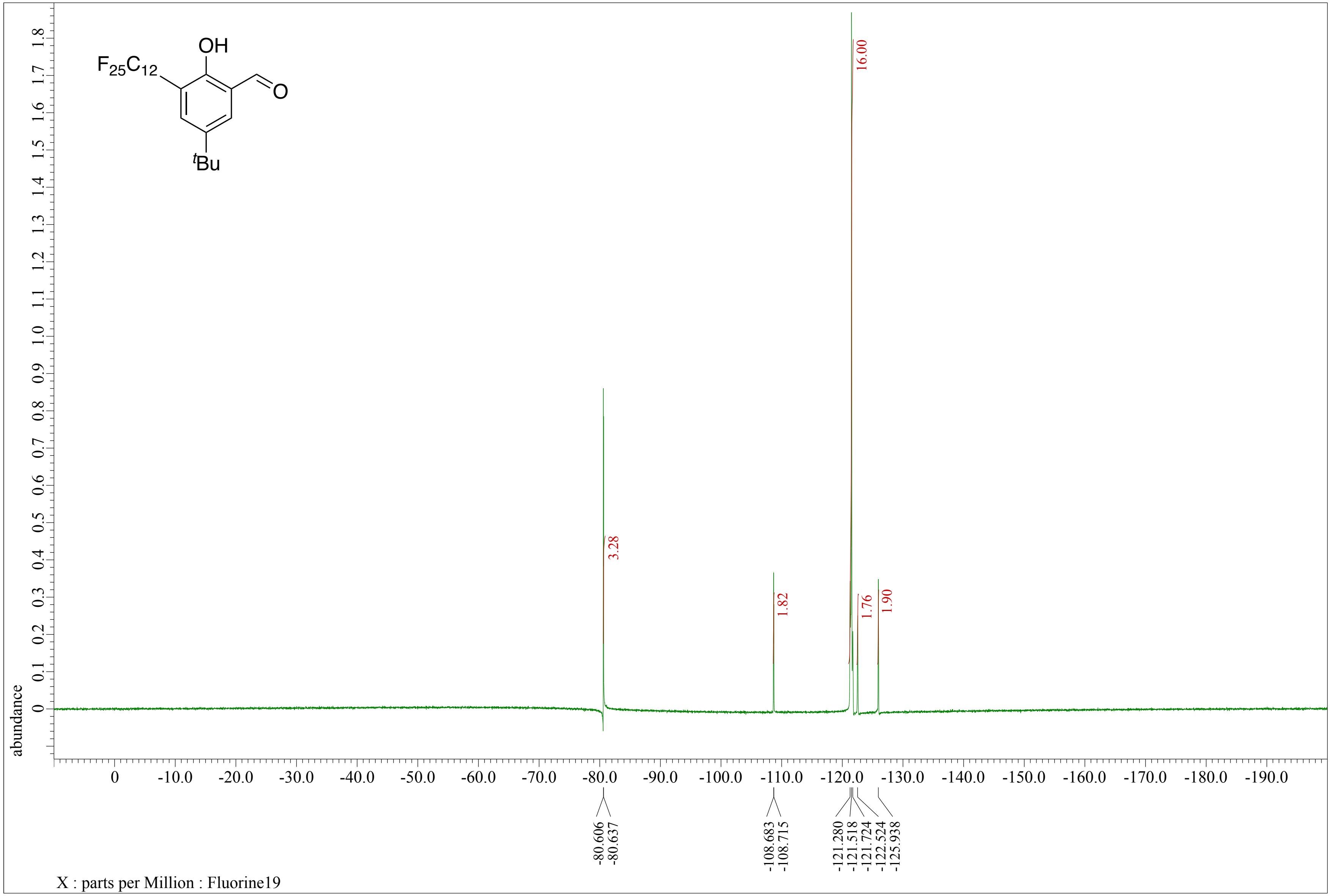

(Ligand of **1b**) ^{1}H NMR, 400 MHz, $CDCl_3$

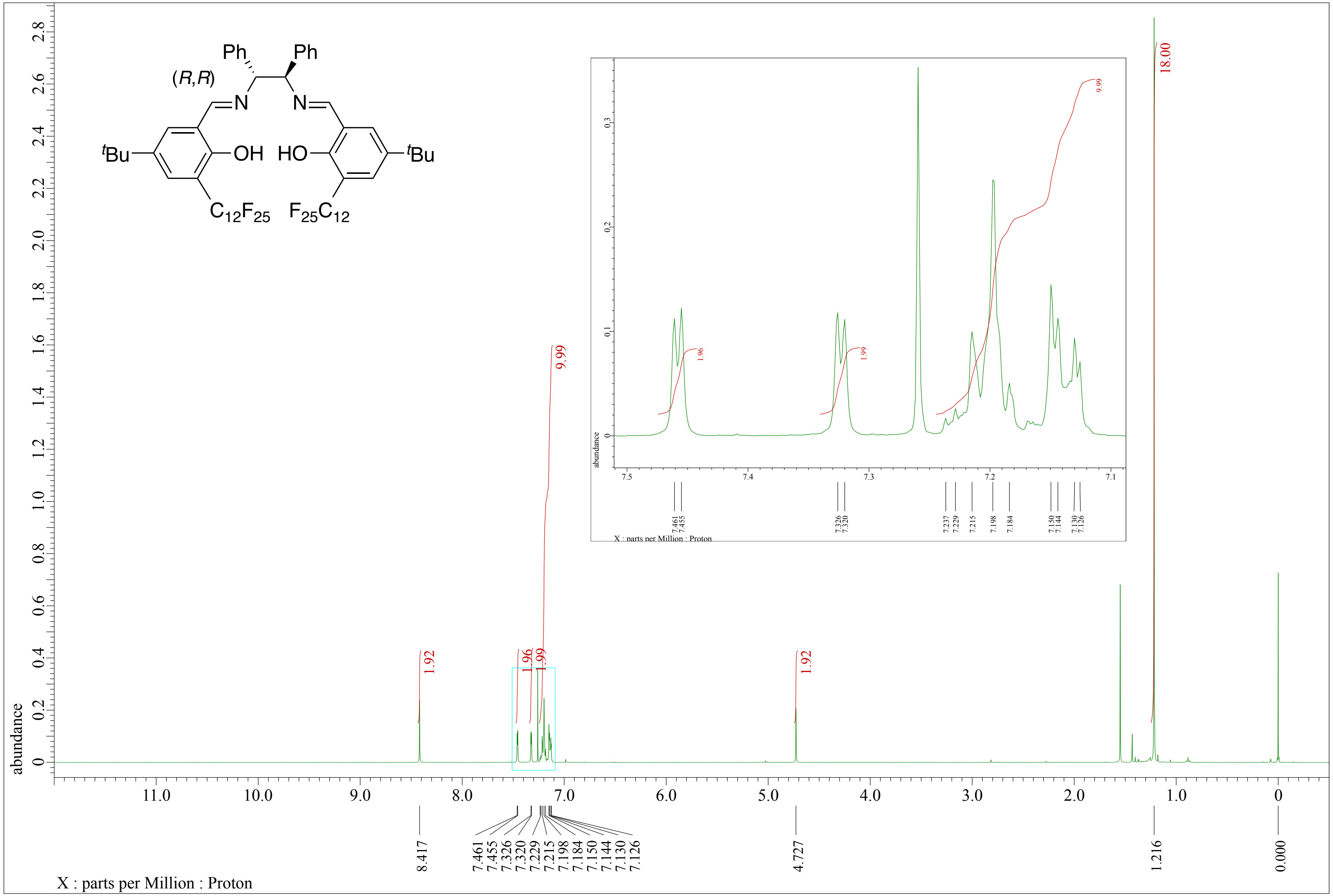

(Ligand of **1b**) ^{13}C NMR, 101 MHz, $CDCl_3$

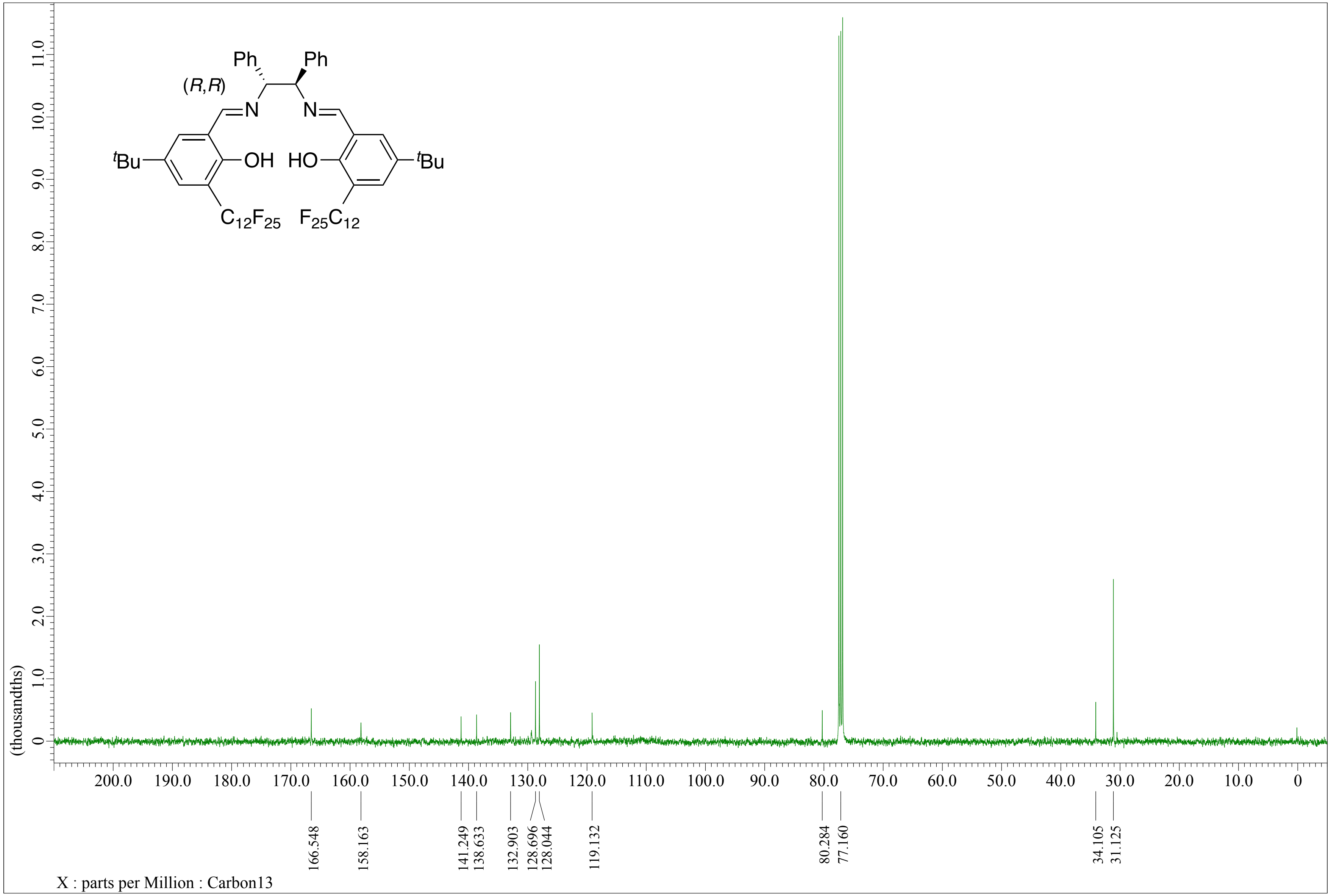

(Ligand of **1b**) ^{19}F NMR, 376 MHz, $CDCl_3$

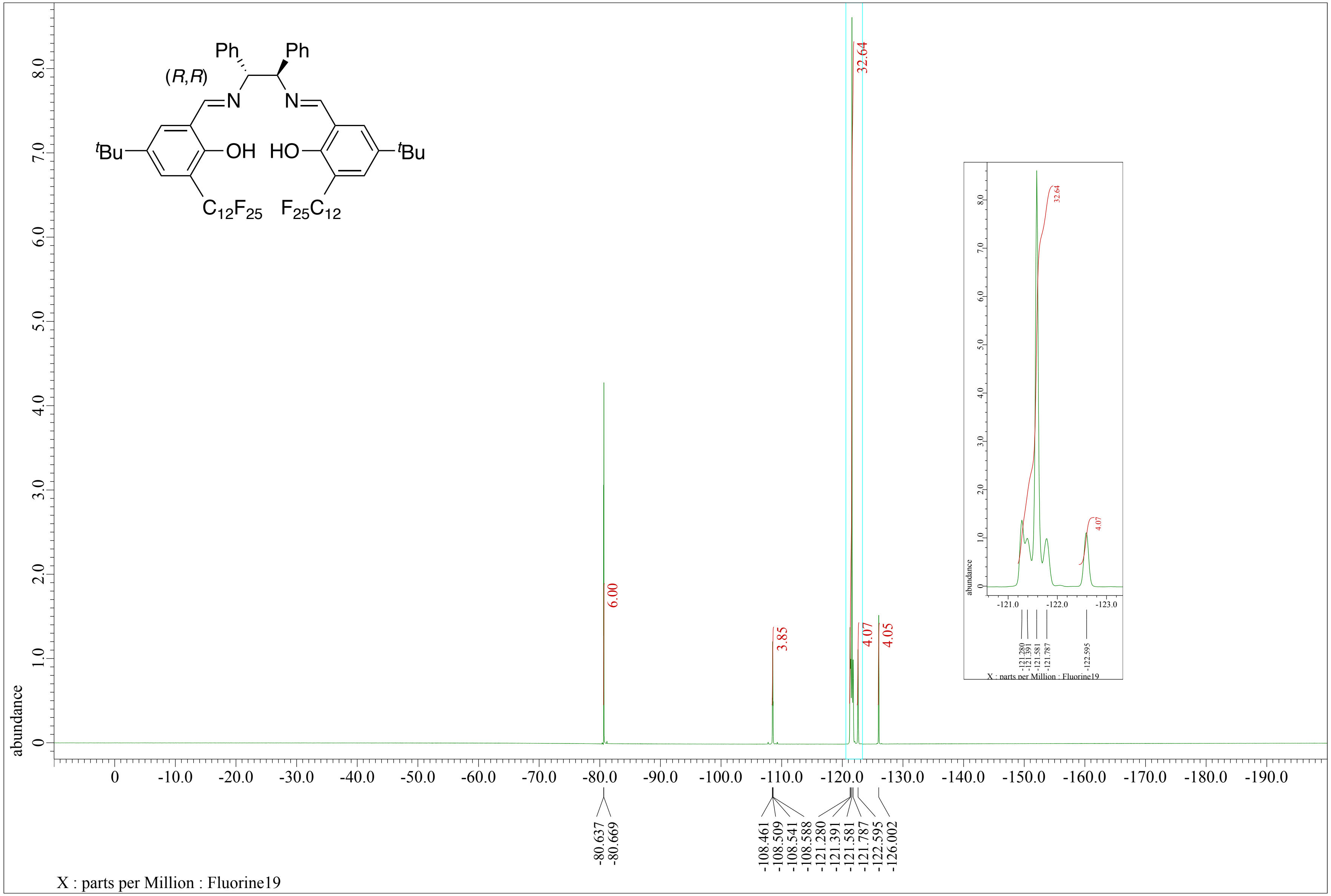

(Ligand of **1c**) ^{1}H NMR, 400 MHz, $CDCl_3$

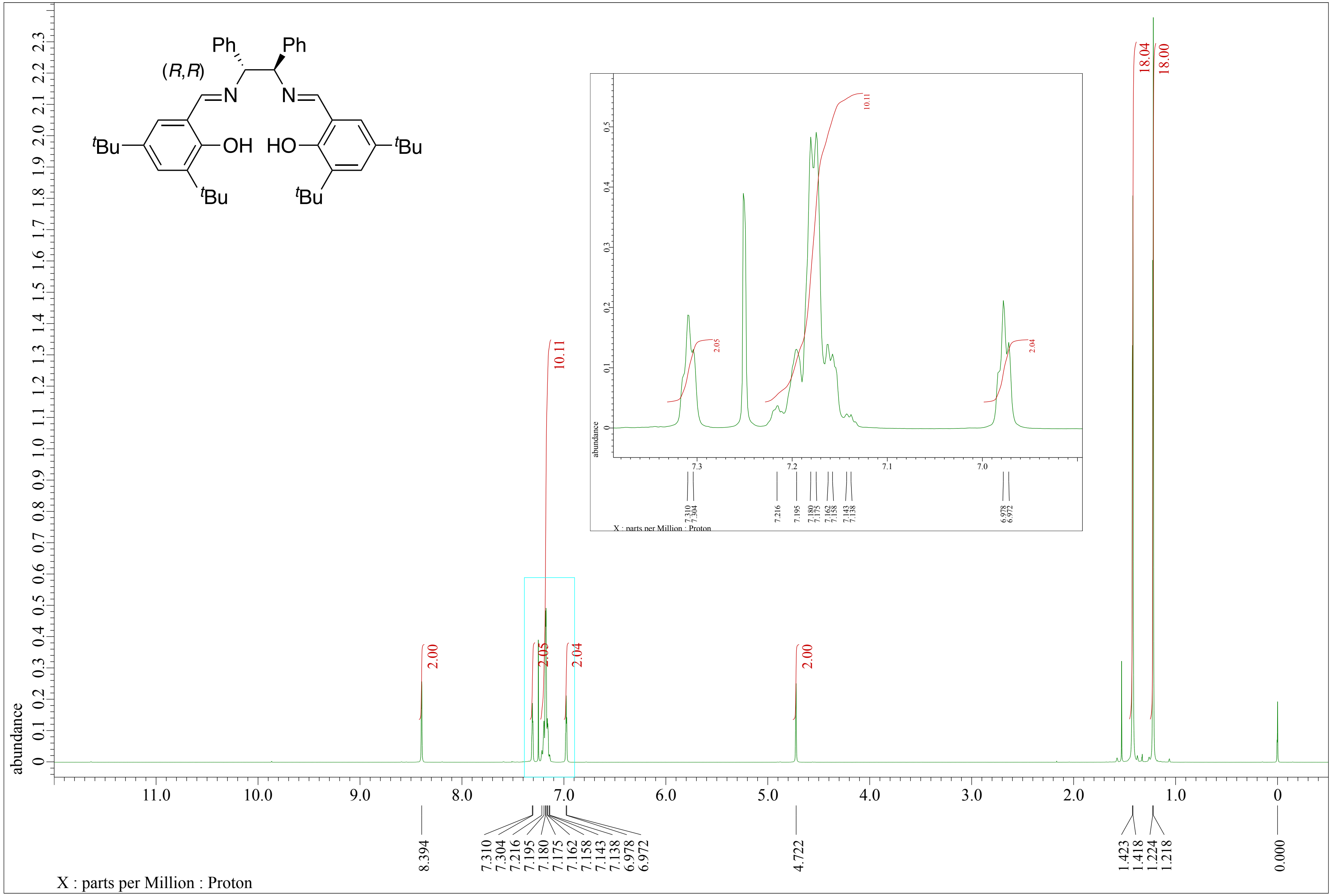

Table 1, **entry 2** ^{1}H NMR, 400 MHz, $CDCl_3$

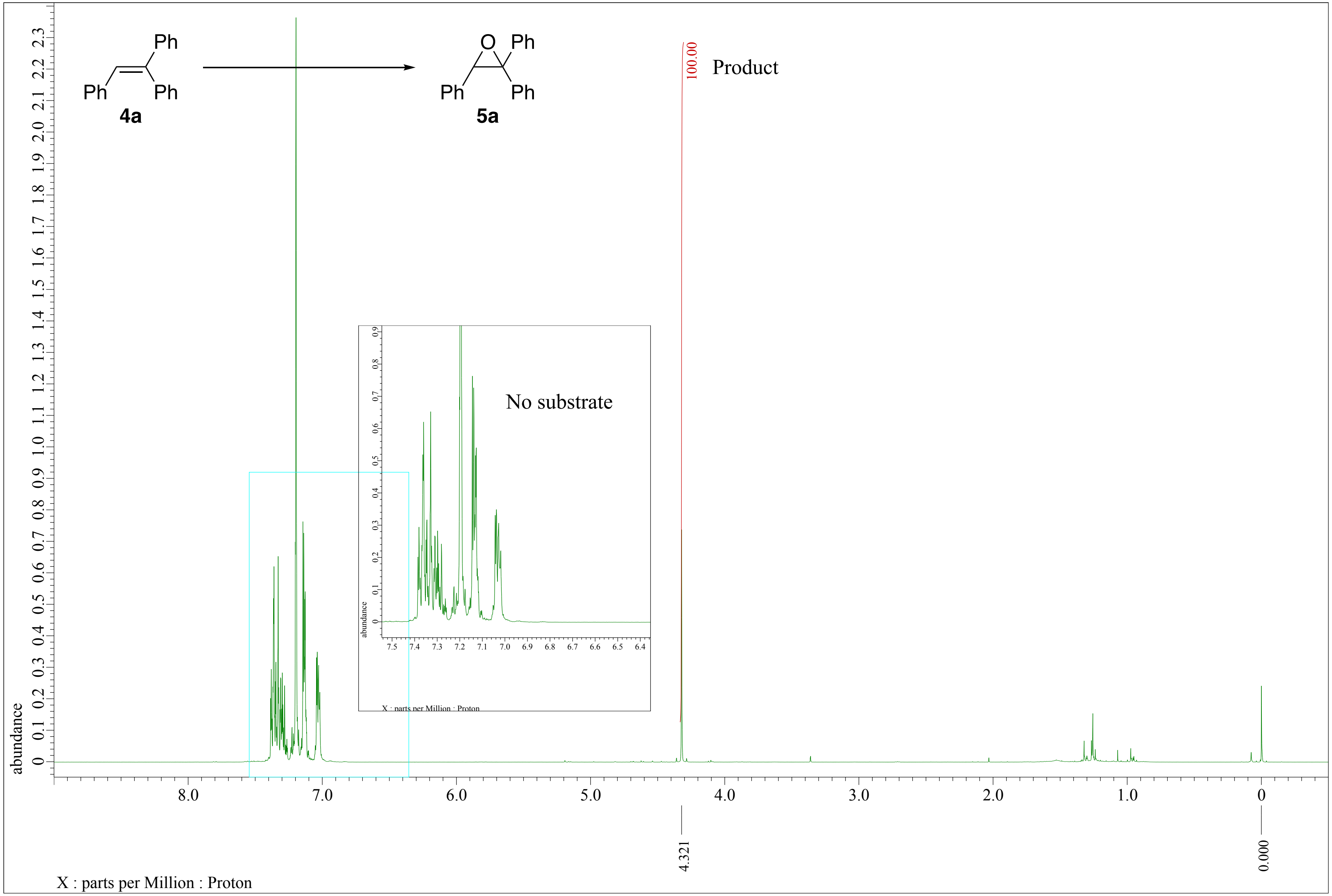

(**5a**) ^{1}H NMR, 400 MHz, $CDCl_3$

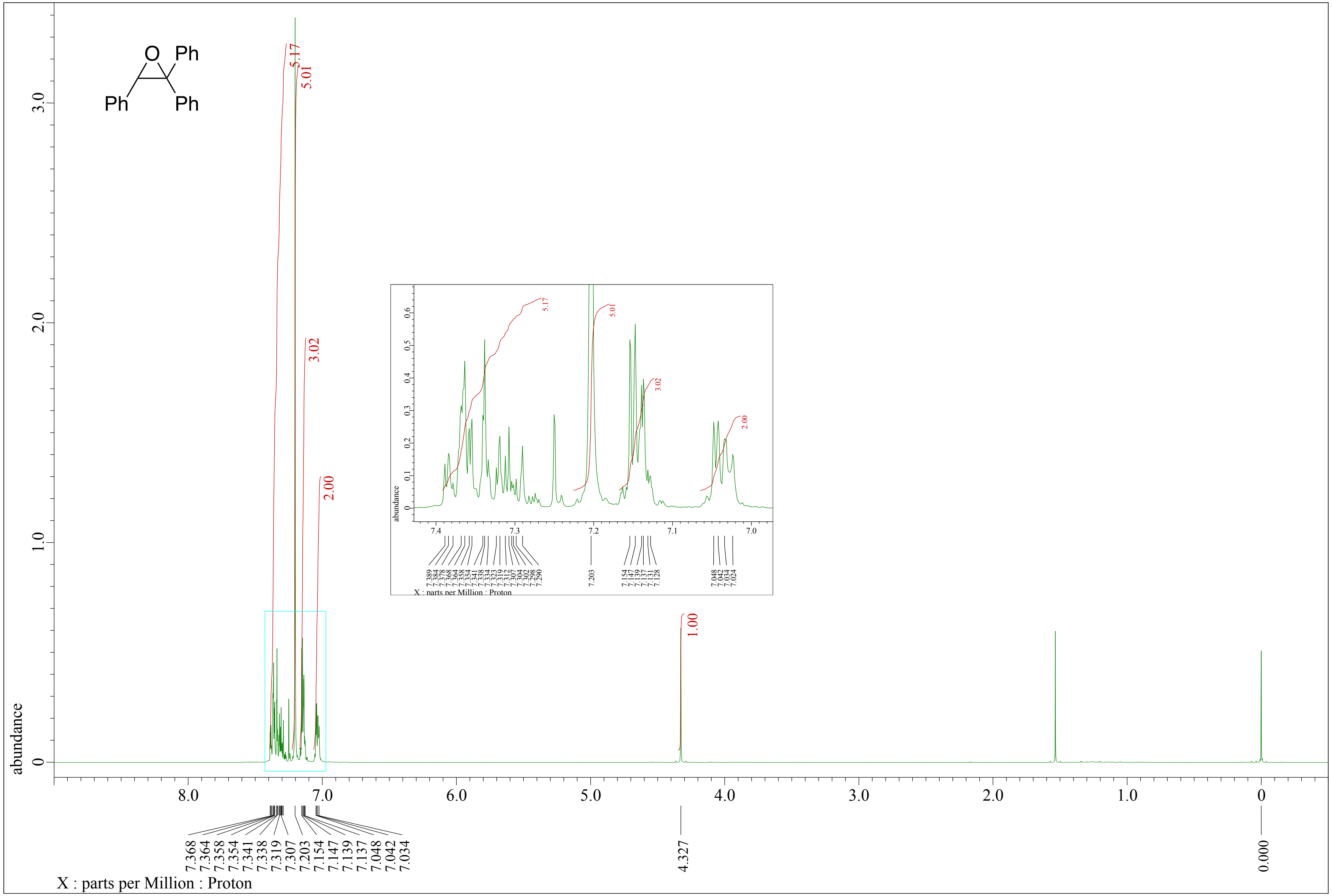

(**4e**) ^{1}H NMR, 400 MHz, $CDCl_3$

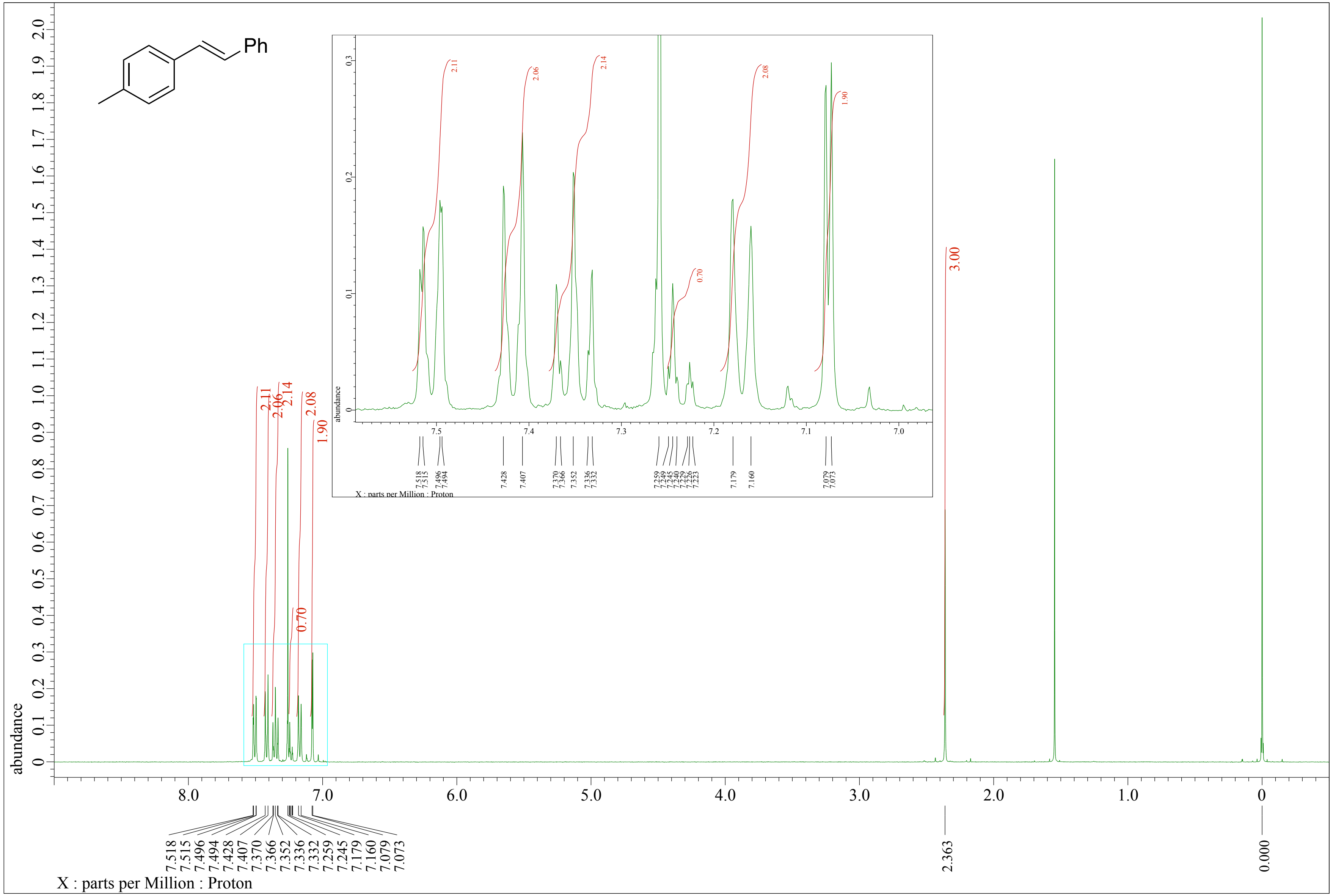

(**4f**) ^{1}H NMR, 400 MHz, $CDCl_3$

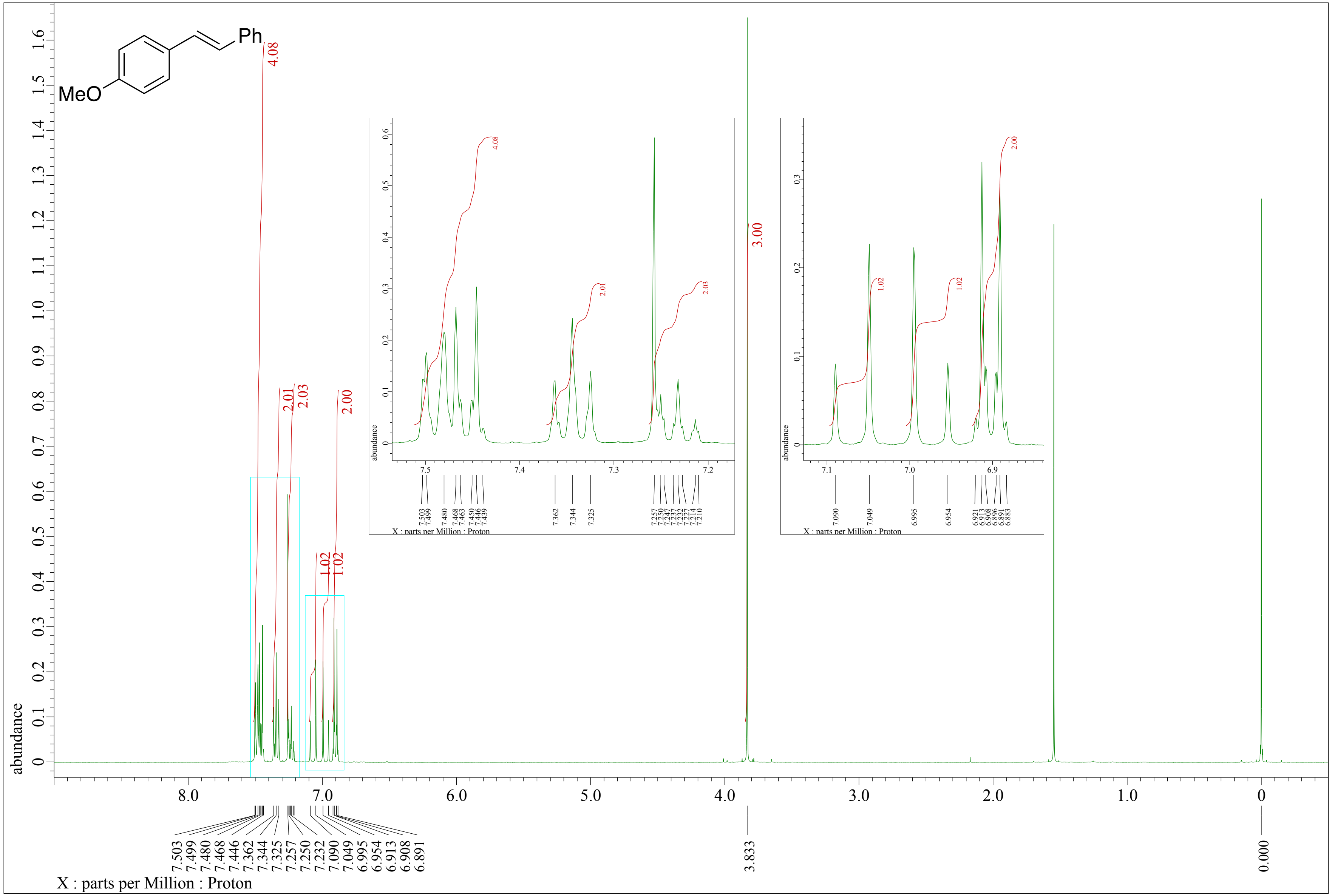

(**4g**) ^{1}H NMR, 400 MHz, $CDCl_3$

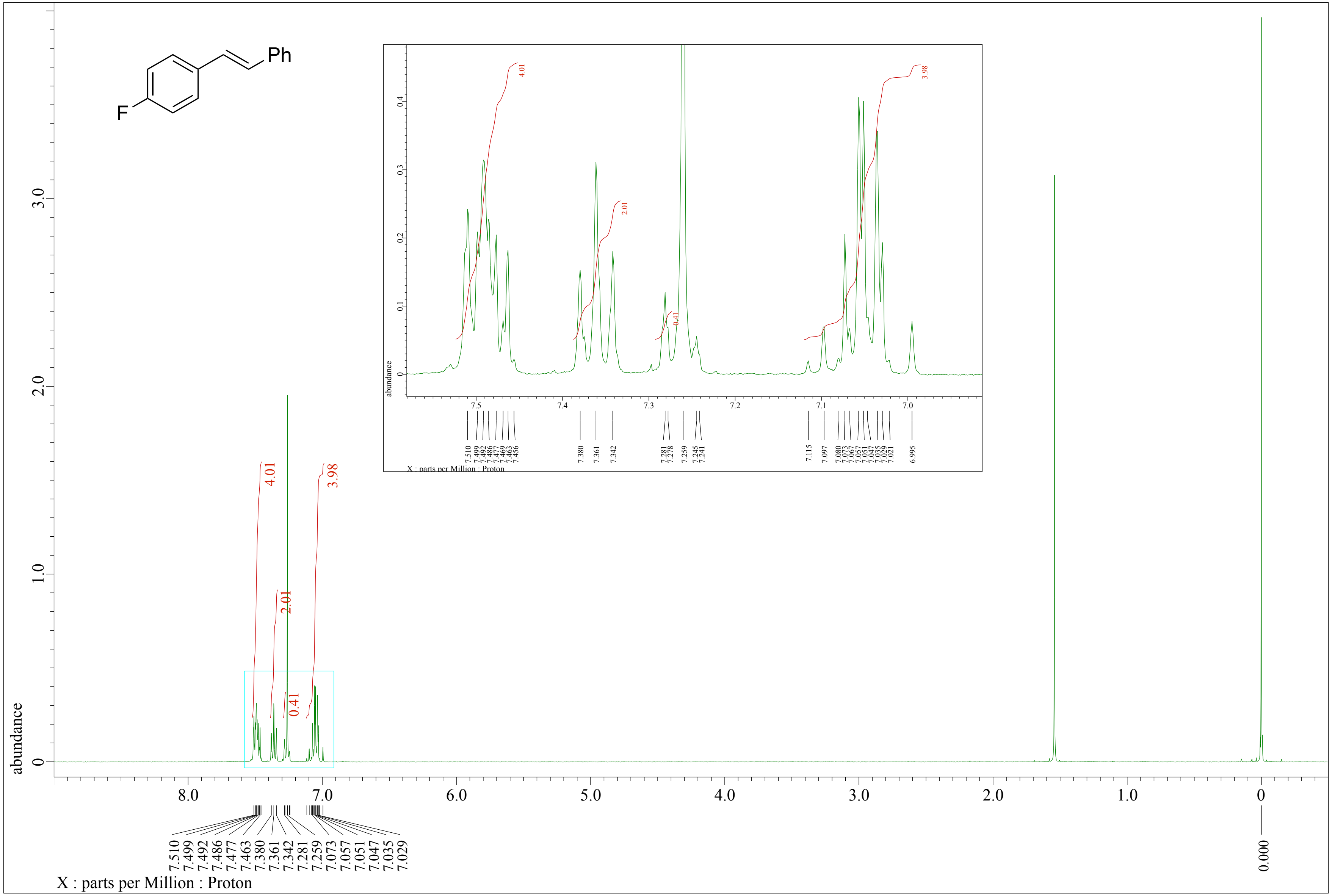

(**4g**) ^{19}F NMR, 376 MHz, $CDCl_3$

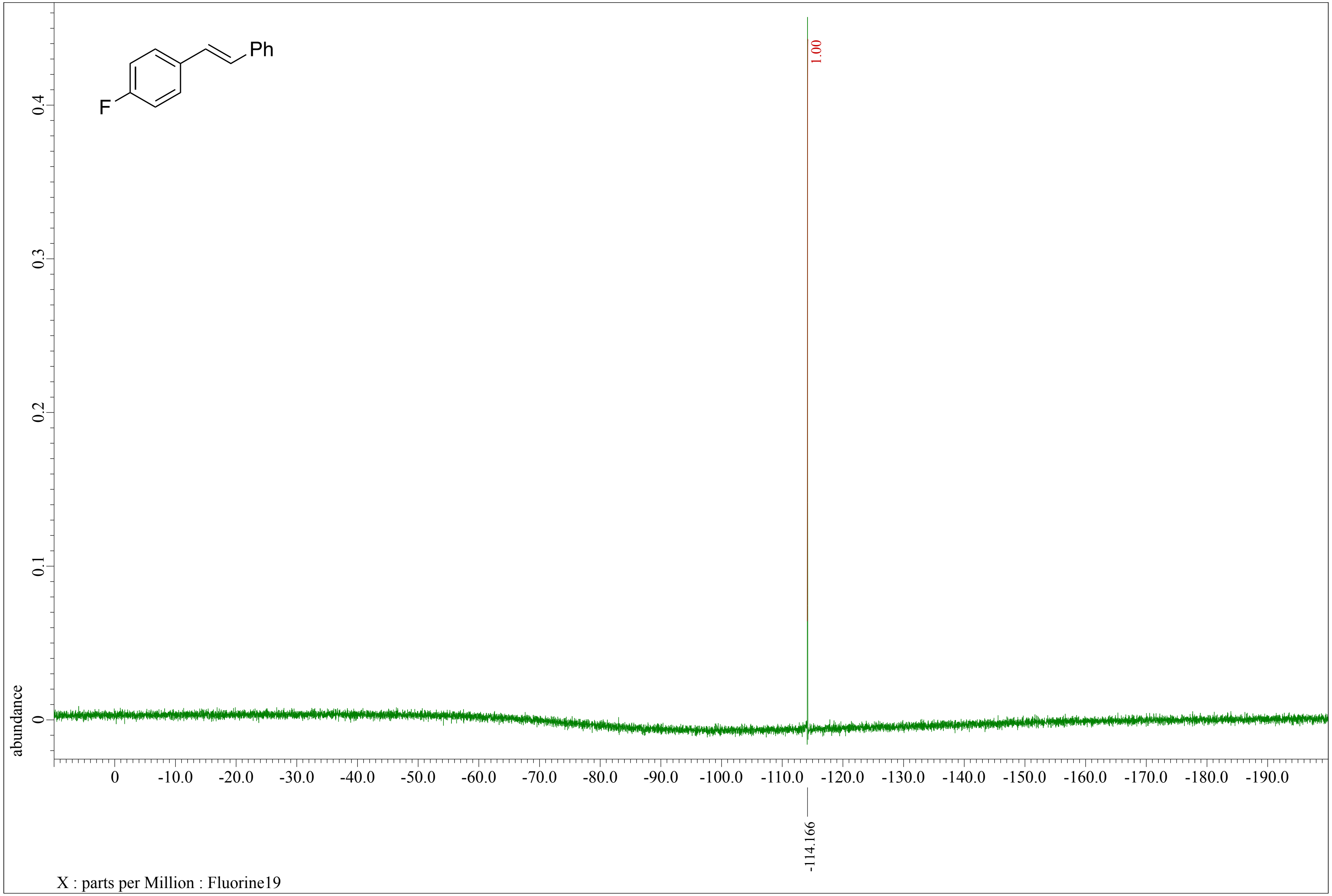

(**4h**) ^{1}H NMR, 400 MHz, $CDCl_3$

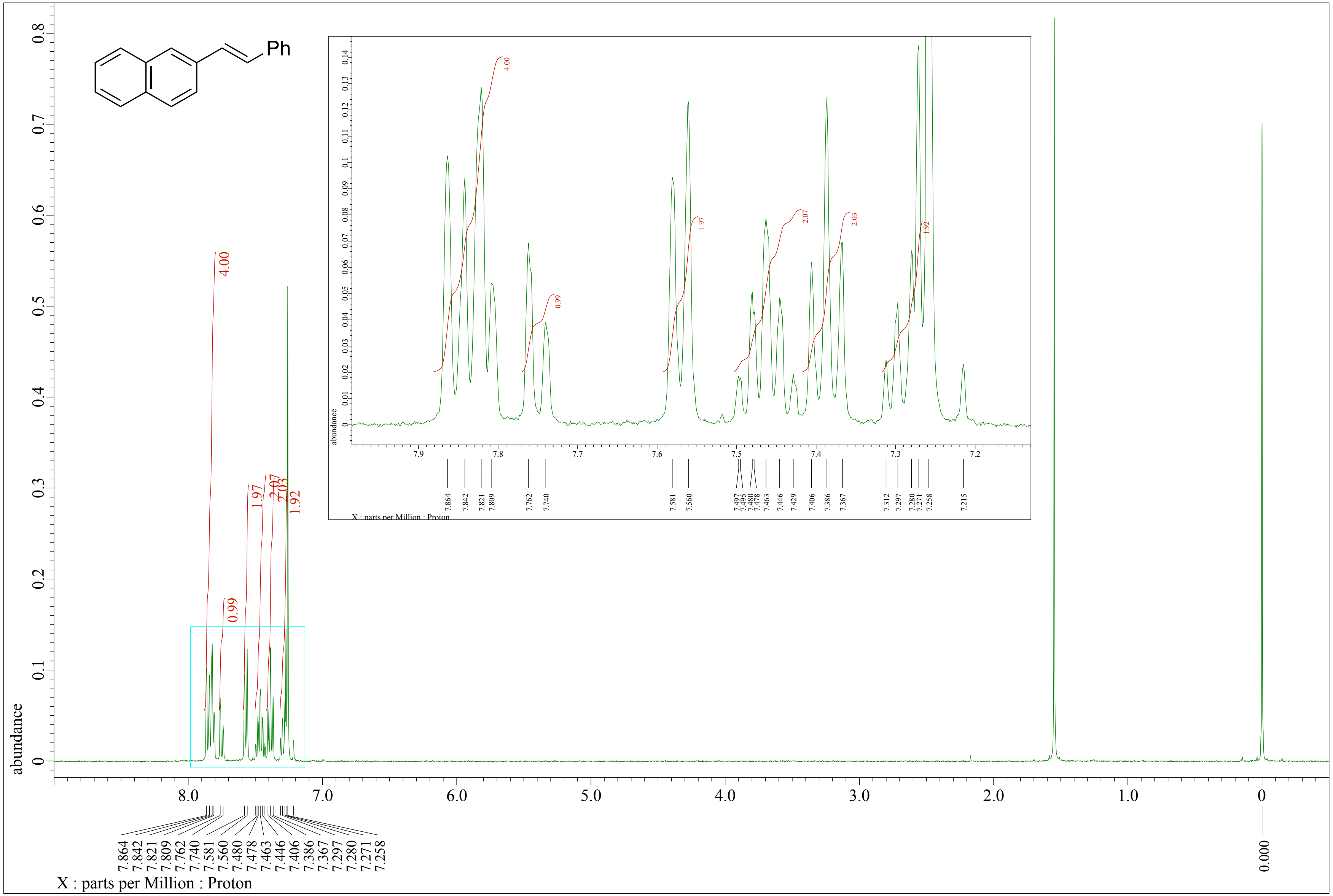

(**4i**) ^{1}H NMR, 400 MHz, $CDCl_3$

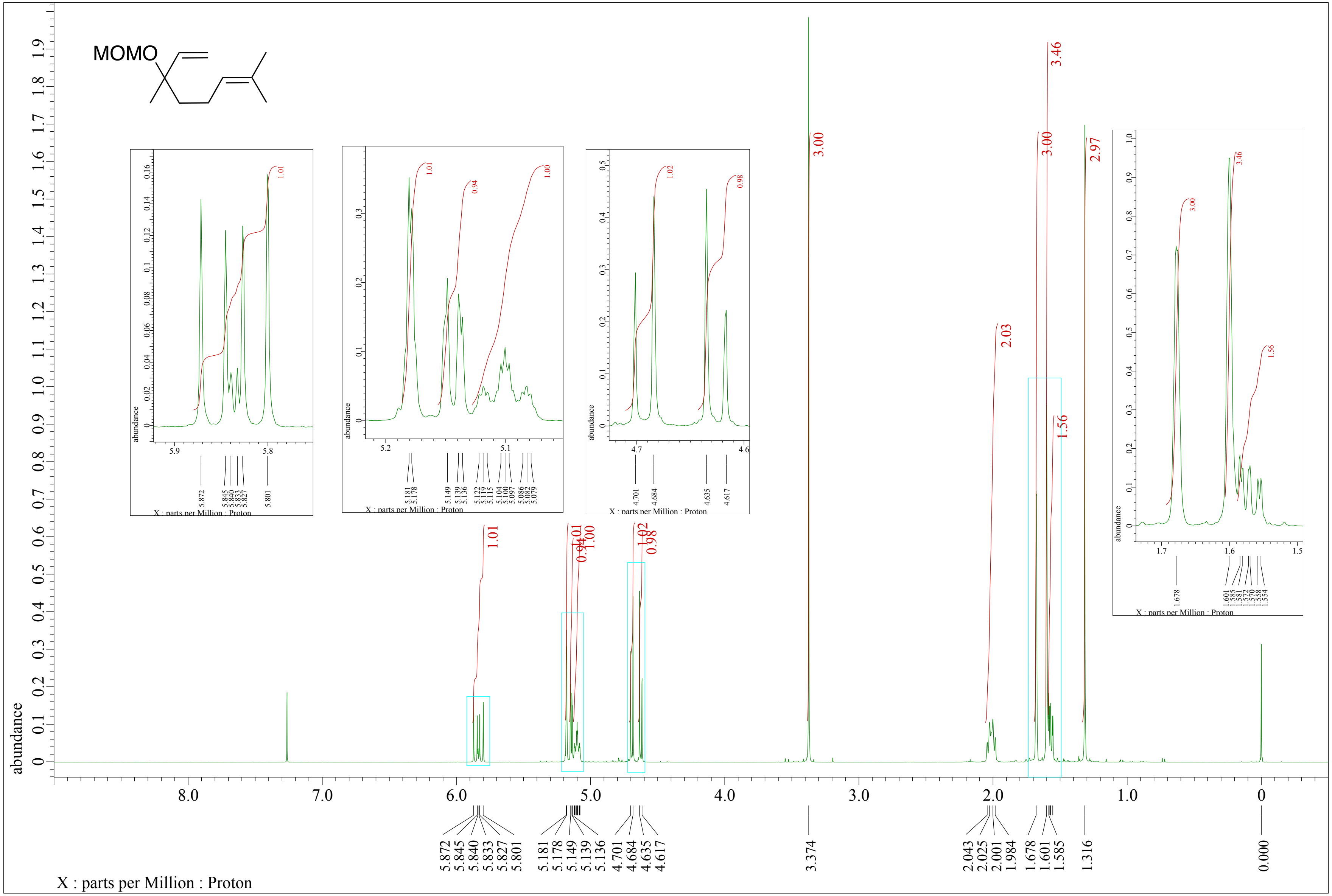

(**4i**) ^{13}C NMR, 101 MHz, $CDCl_3$

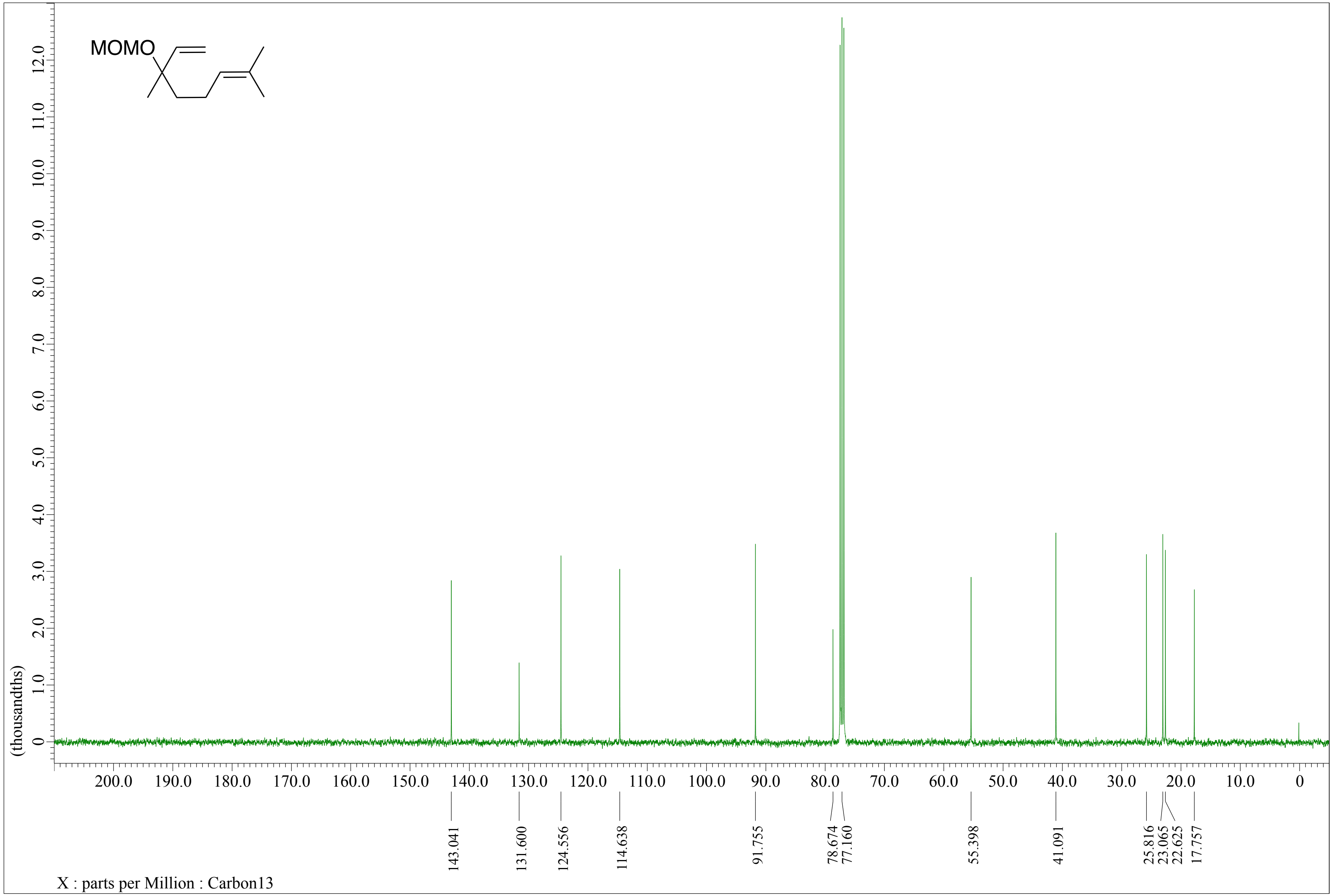

(**5b**) ^{1}H NMR, 400 MHz, $CDCl_3$

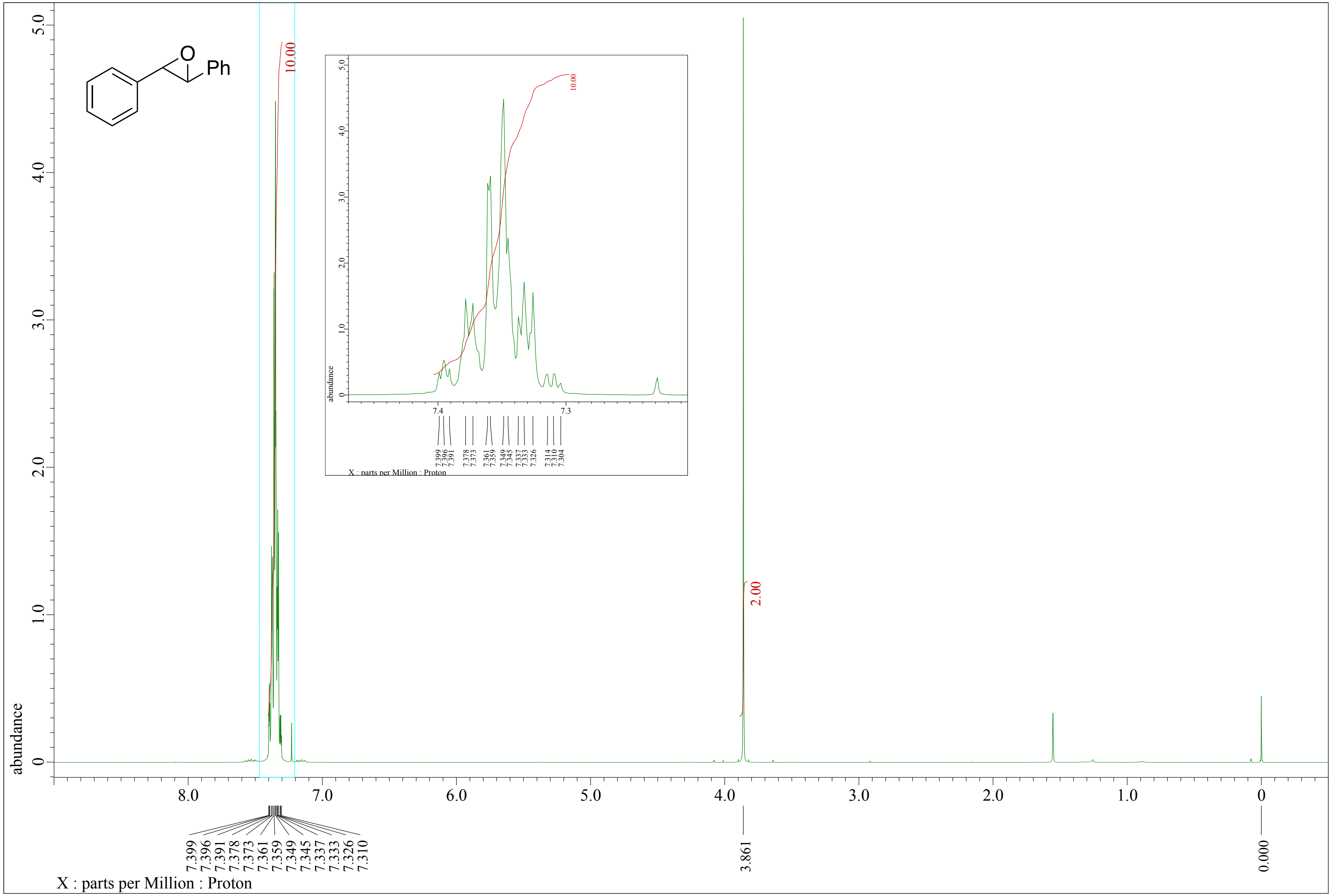

(**5c**) ^{1}H NMR, 400 MHz, $CDCl_3$

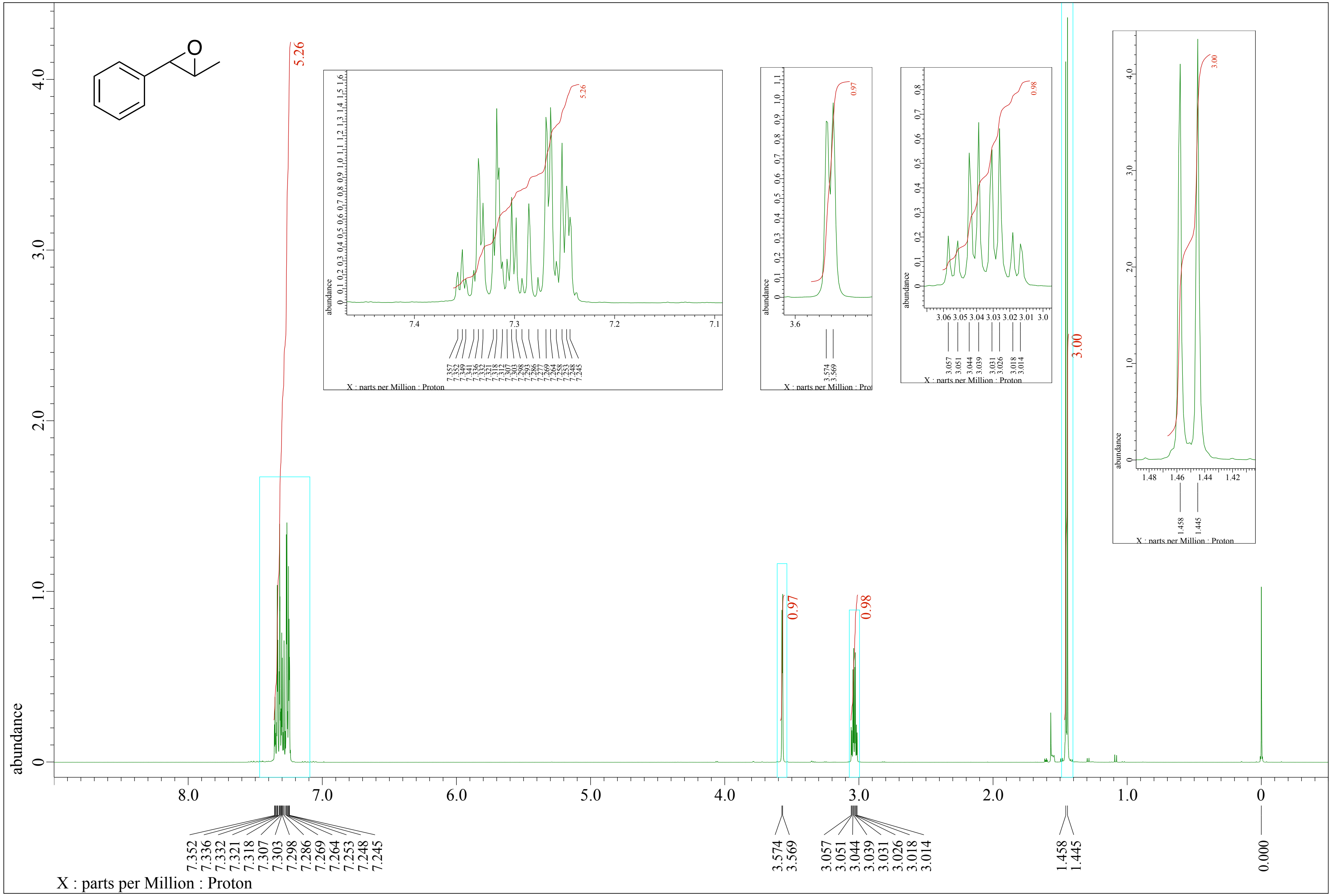

(**5d**) ^{1}H NMR, 400 MHz, $CDCl_3$

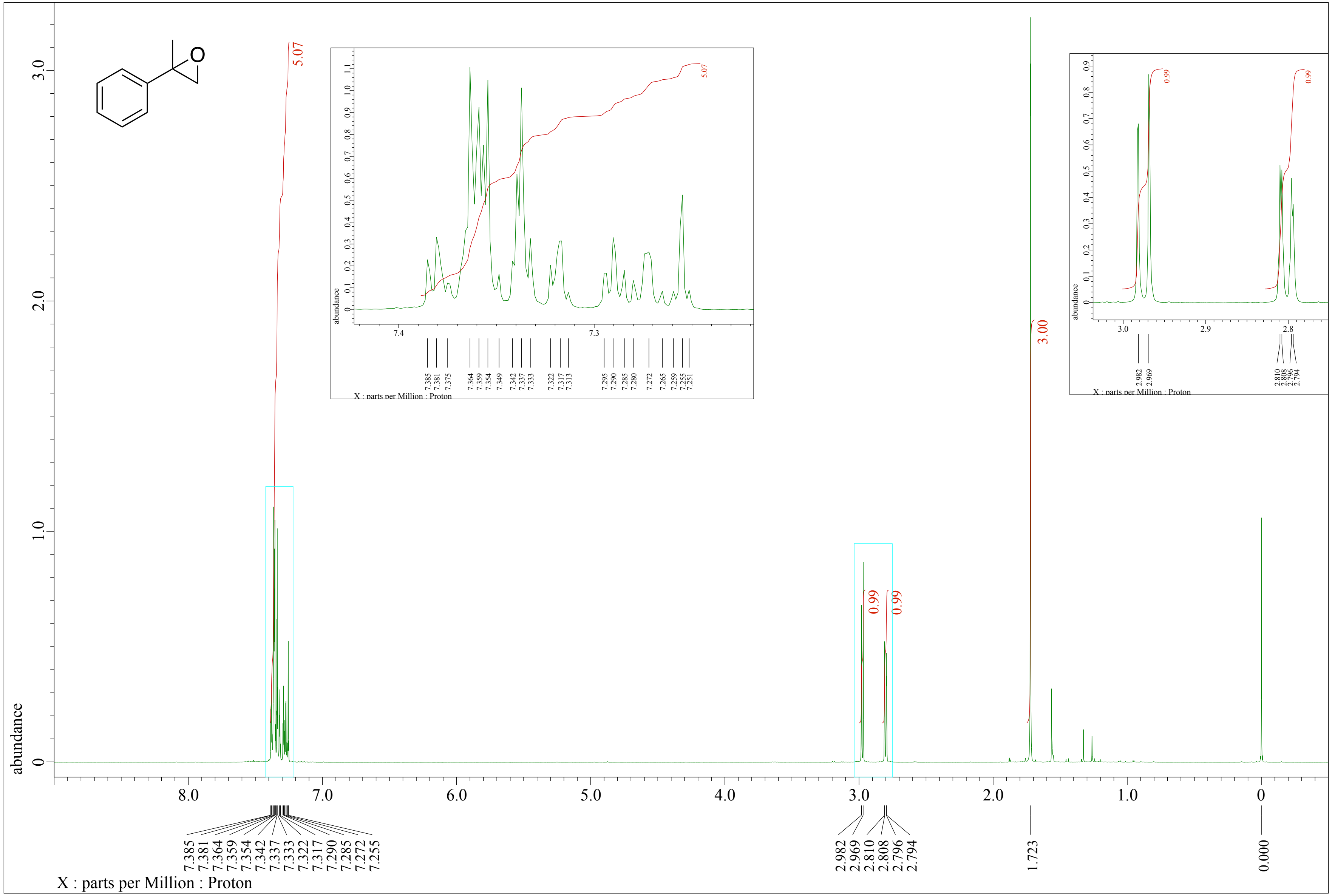

(**5e**) ^{1}H NMR, 400 MHz, $CDCl_3$

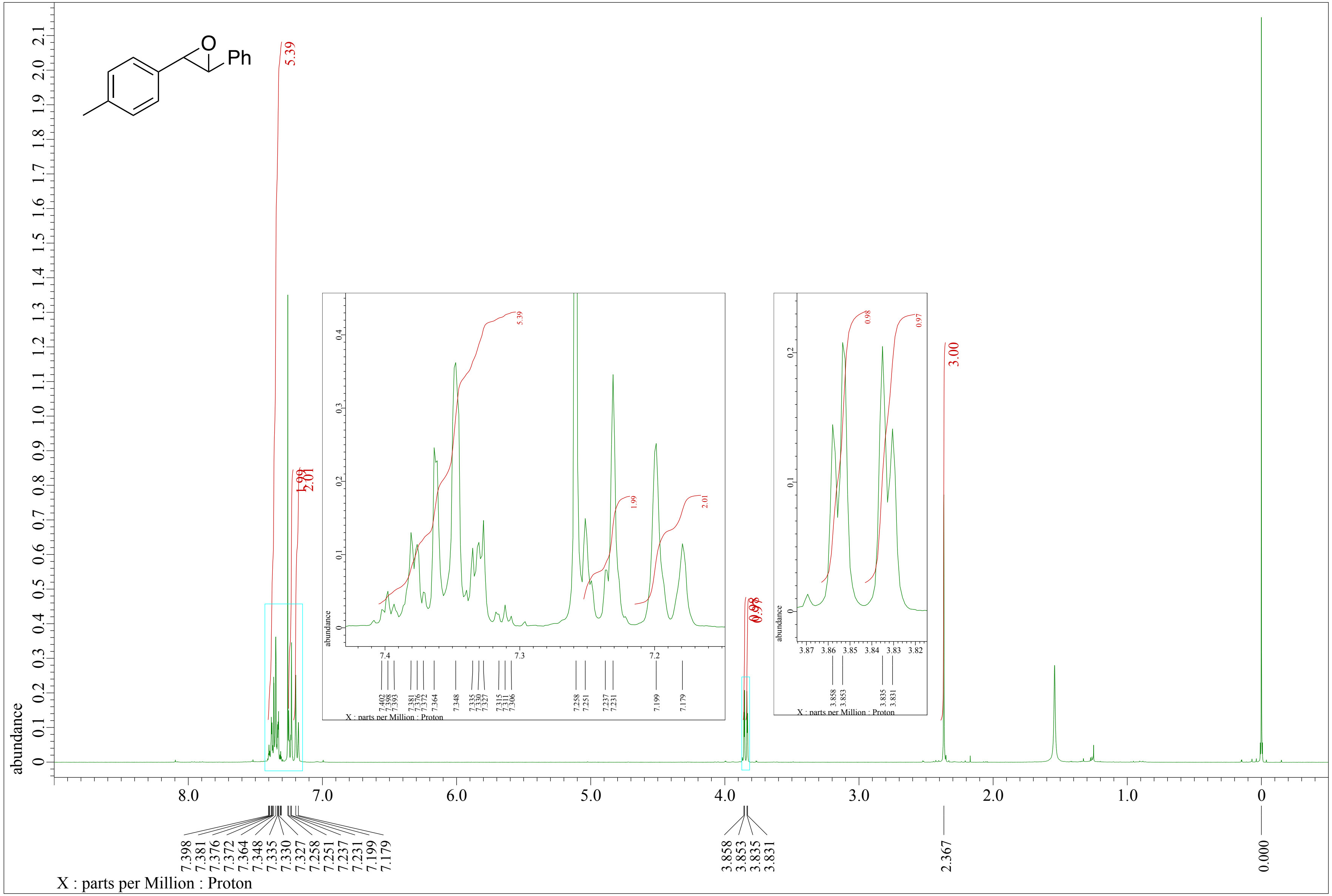

(**5f**) ^{1}H NMR, 400 MHz, $CDCl_3$

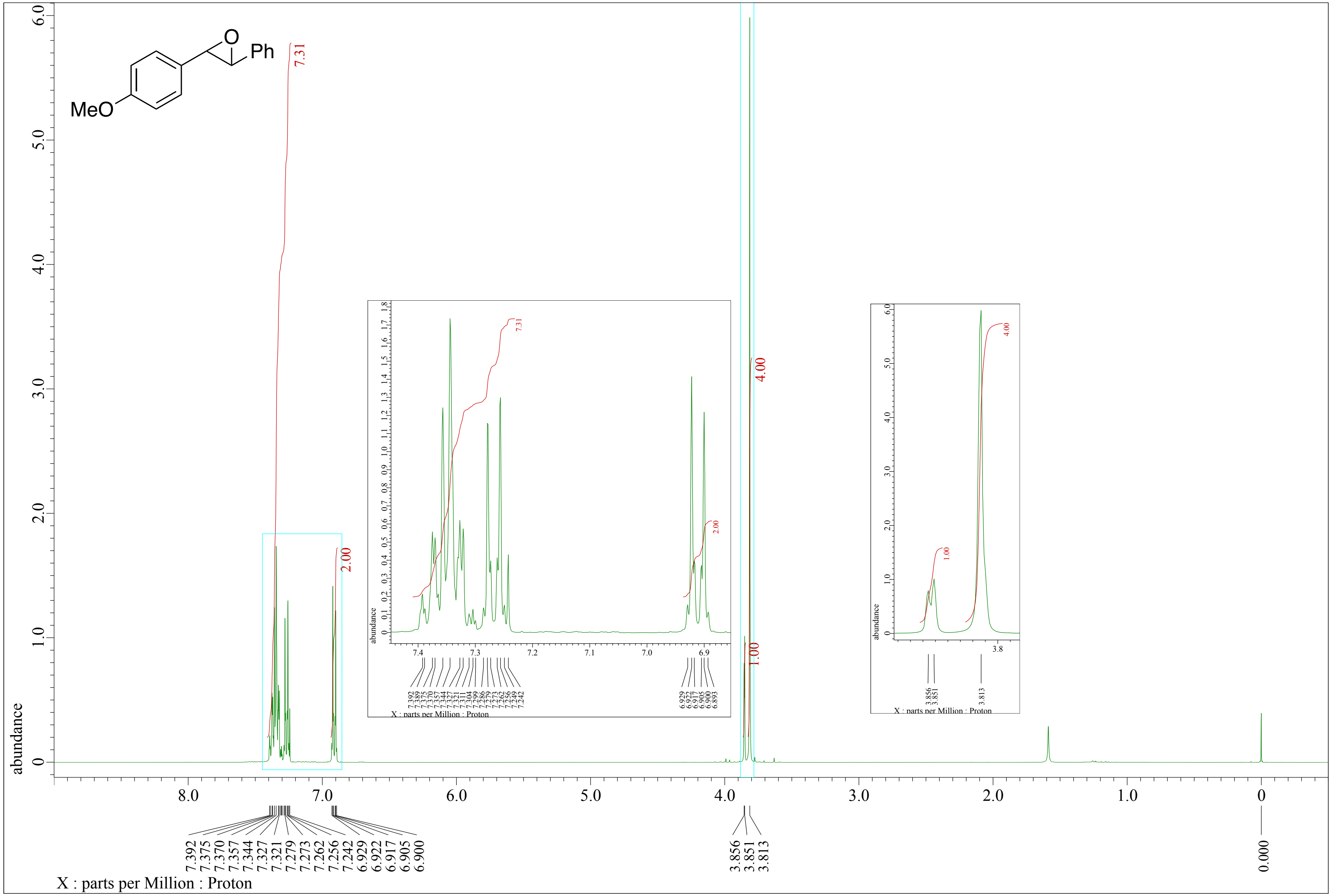

(**5g**) ^{1}H NMR, 400 MHz, $CDCl_3$

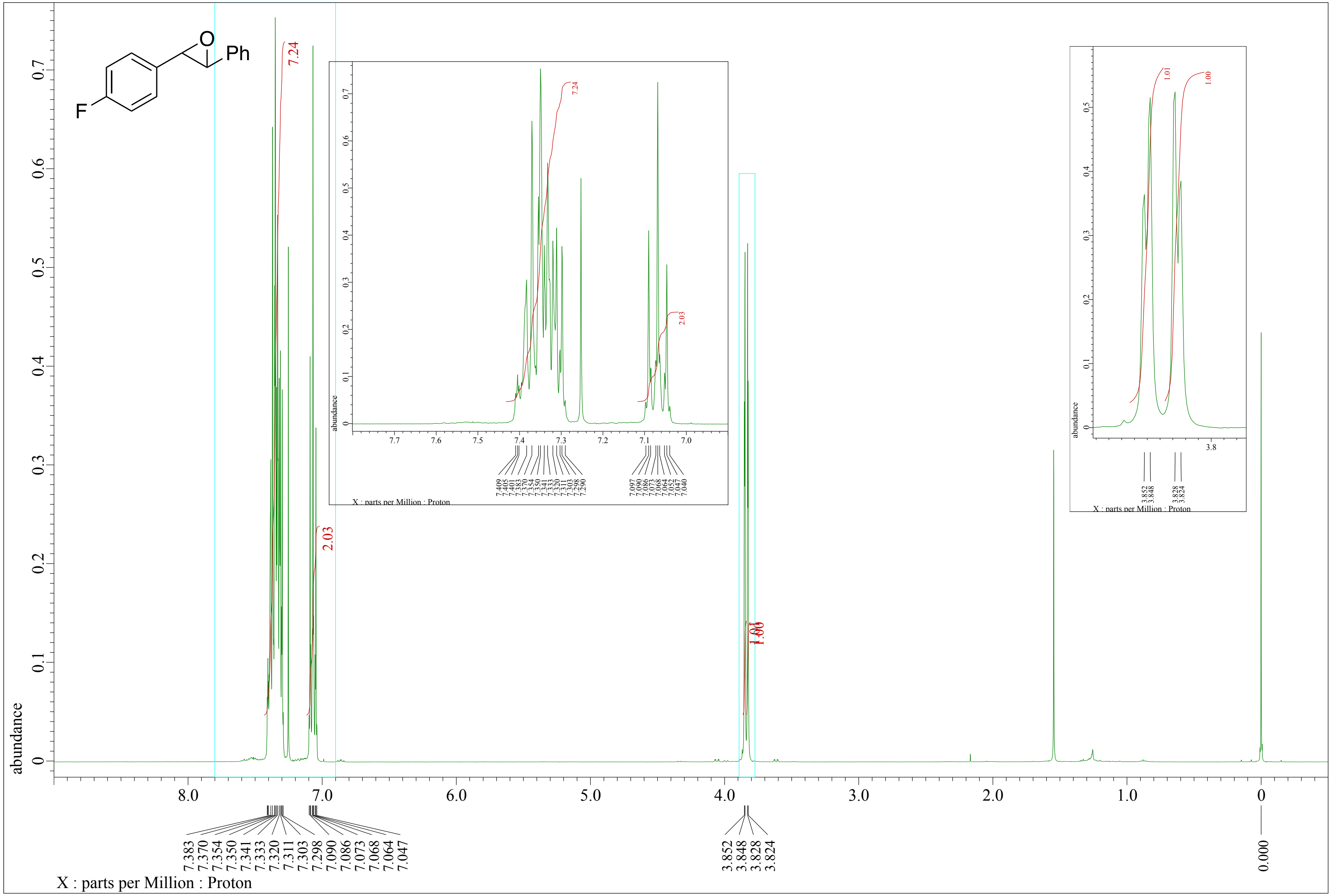

(**5g**) ^{19}F NMR, 376 MHz, $CDCl_3$

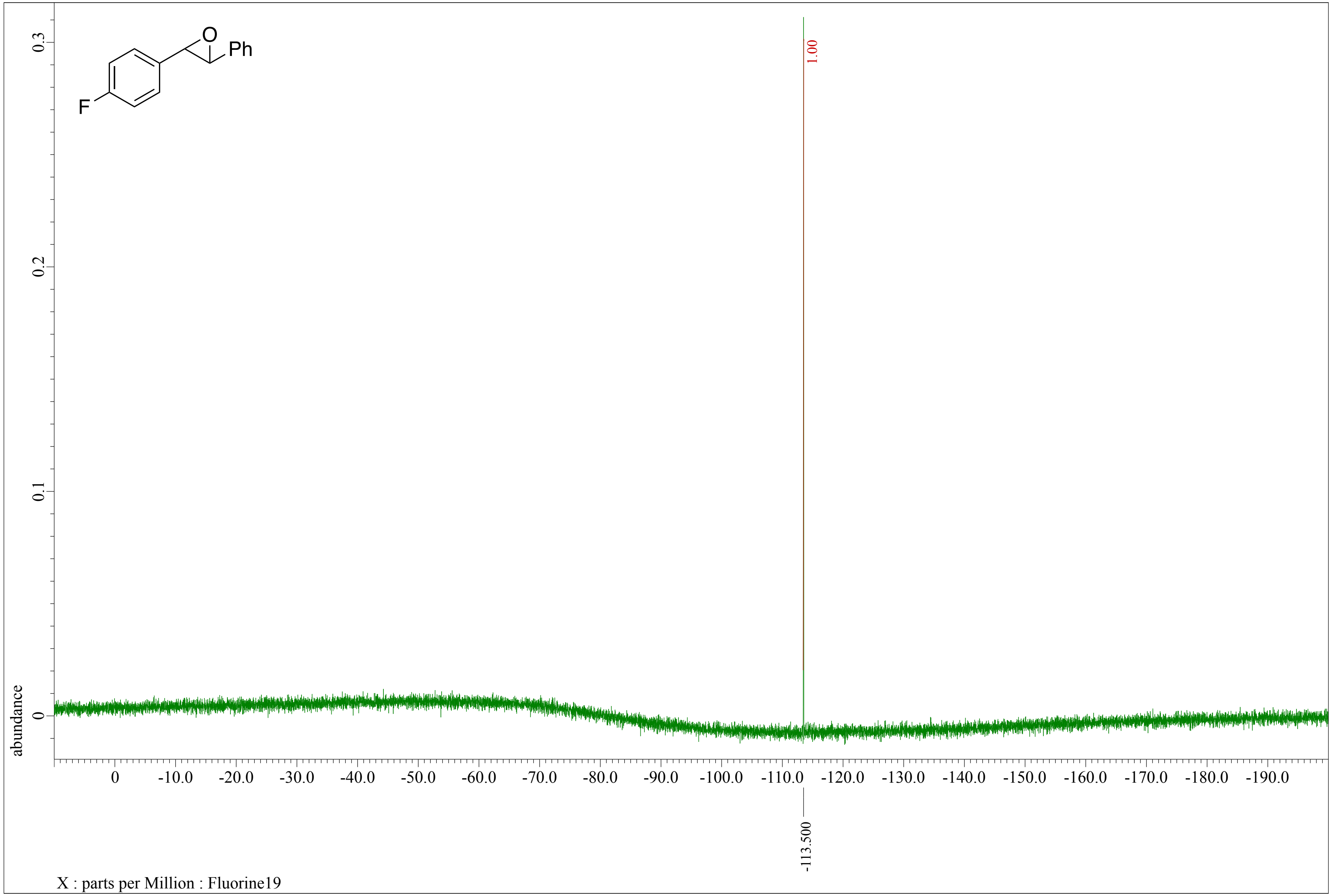

(**5h**) ^{1}H NMR, 400 MHz, $CDCl_3$

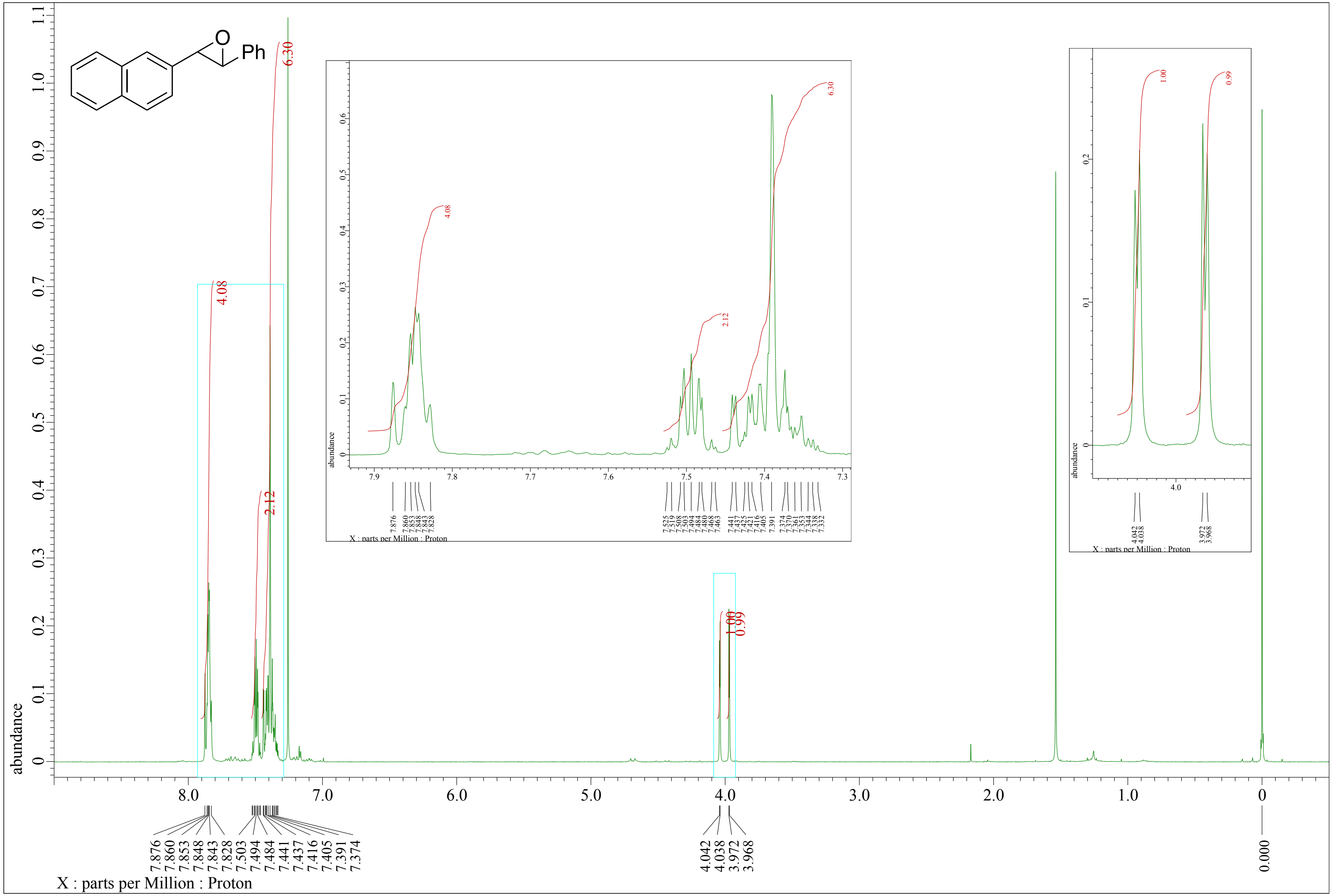

(**5i**) ^{1}H NMR, 400 MHz, $CDCl_3$

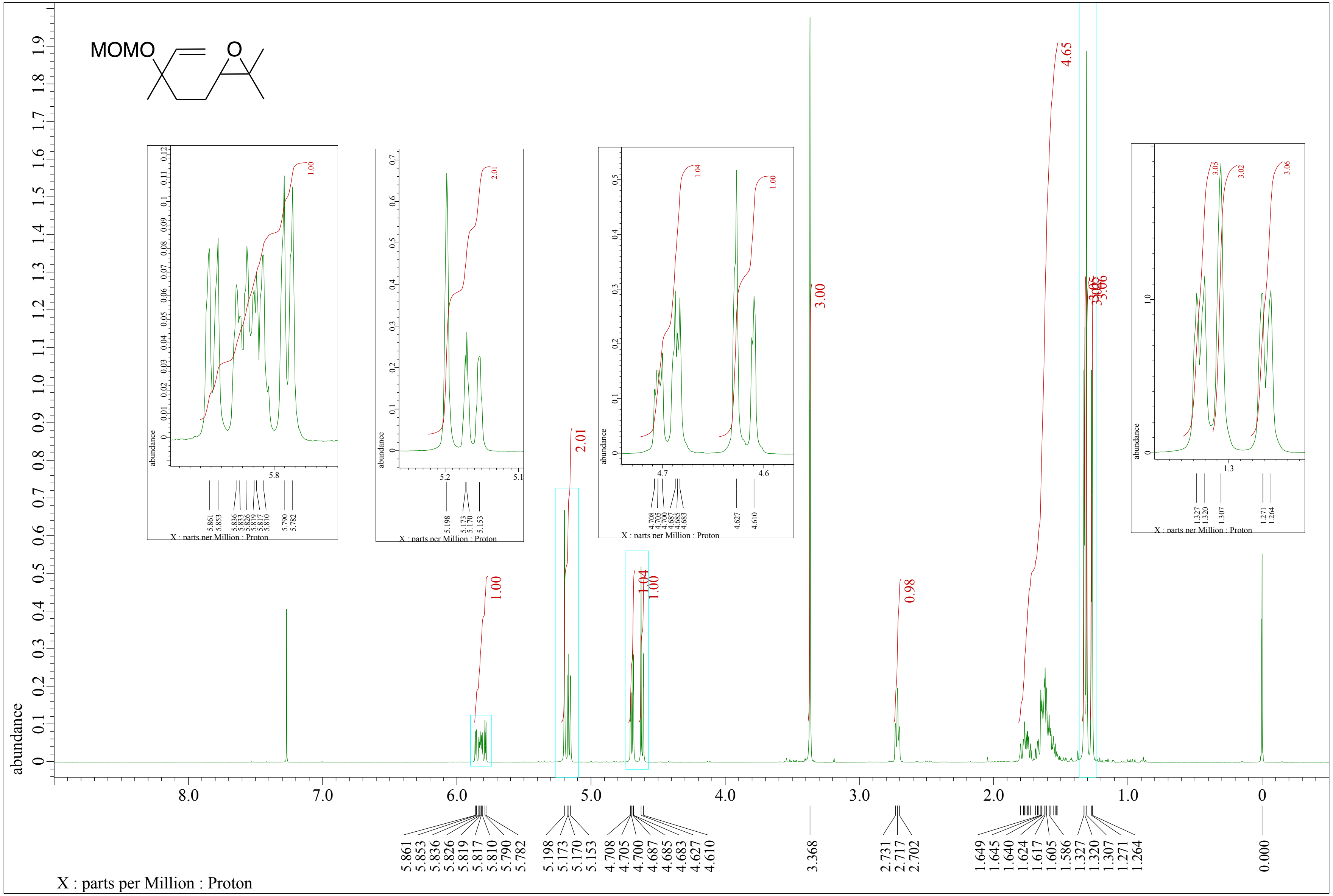

(**5i**) ^{13}C NMR, 101 MHz, $CDCl_3$

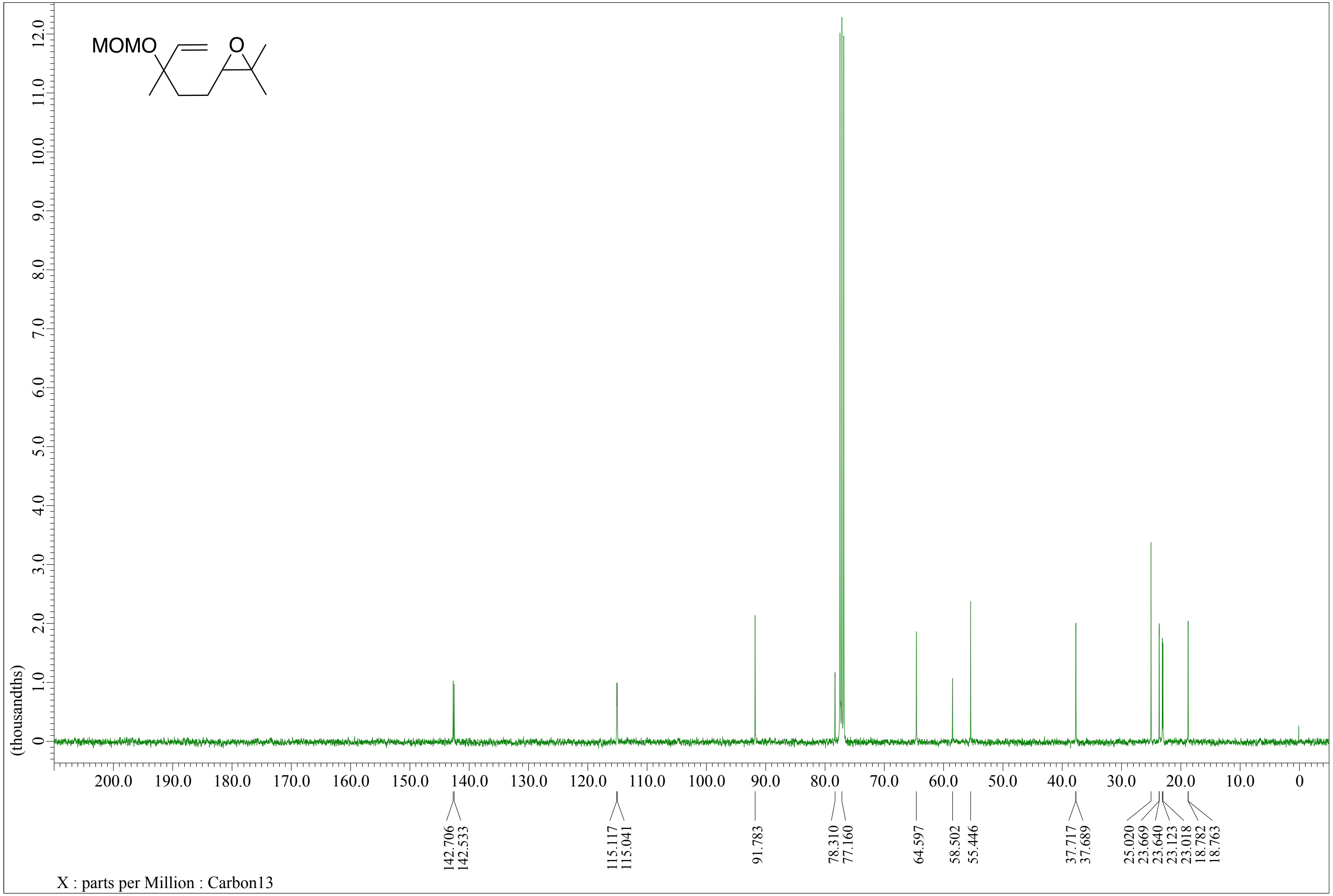

Supplement: Supplementary file 1 [file molecules-29-00966-s001.zip › molecules-2871253-supplementary.pdf]
